# Social environments and lifestyles linked to mortality and future health risks in metabolic syndrome: evidence from UK and US nationwide cohort studies

**DOI:** 10.1136/bmjph-2025-004599

**Published:** 2026-08-05

**Authors:** Qiuyue Dong, Xiao Yin, Zhengyue Jing, Alimu Dayimu, Tiantian Gao, YiLin Gai, Yunfei Li, Shanquan Chen, Fan Jiang, Liang Wang

**Affiliations:** 1 School of Public Health, Shandong Provincial ENT Hospital, Shandong University, Jinan, Shandong, China; 2 Department of Endocrinology, China-Japan Friendship Hospital, Beijing, China; 3 Department of Social Security, School of Health Policy and Management, Nanjing Medical University, Nanjing, China; 4 Cambridge Clinical Trials Unit Cancer Theme, University of Cambridge, Cambridge, UK; 5 Department of Pharmacy, Shandong Provincial Hospital Affiliated to Shandong First Medical University, Jinan, China; 6 Centre for Health Management and Policy Research, School of Public Health, Cheeloo College of Medicine, Shandong University, Jinan, Shandong, China; 7 School of Public Health, Capital Medical University, Beijing, China; 8 Department of Neurobiology, Care Sciences and Society, Karolinska Institutet, Stockholm, Sweden; 9 School of Public Health, Li Ka Shing Faculty of Medicine, The University of Hong Kong, Hong Kong, Hong Kong; 10 Department of Health and Rehabilitation Sciences, Faculty of Medicine and Health Sciences, Stellenbosch University, Stellenbosch, South Africa; 11 Department of Public Health, College of Health Professions; Global Health Institute, Marshall University, Huntington, West Virginia, USA

**Keywords:** Public Health, Community Health, Public Health Practice

## Abstract

**Introduction:**

Metabolic syndrome (MetS) poses a major public health challenge. This study examined how social environments and their interactions with lifestyle behaviours influence mortality, life expectancy and disease risks in adults with MetS from the UK and USA.

**Methods:**

Adults with MetS were identified from the UK Biobank (2006–2010, n=60 423; mean follow-up 12.8 years) and US National Health and Nutrition Examination Survey (NHANES) (1999–2018, n=11 170; mean follow-up 8.0 years). Weighted scores for social environment (socioeconomic status, social capital, healthcare access, built environment) and healthy lifestyle (smoking, alcohol, physical activity, diet) were categorised into tertiles. Primary outcomes were all-cause and cause-specific mortality (cardiovascular disease (CVD), cerebrovascular disease (CBVD), chronic kidney disease (CKD)) in both cohorts and incident diseases in UK Biobank. Cox regression assessed associations and interactions.

**Results:**

Unfavourable (vs favourable) social environments were associated with higher all-cause mortality in UK Biobank (HR 1.72, 95% CI 1.62 to 1.83) and NHANES (HR 2.30, 95% CI 1.95 to 2.71), with consistent patterns for CVD, CBVD and CKD outcomes. Life expectancy at age 45 was reduced by 3.49 (95% CI 3.40 to 3.57) years (UK Biobank) and 7.81 (95% CI 7.56 to 8.07) years (NHANES). Social environments contributed substantially to mortality burden (population attributable fraction: 17.0% and 24.1%, respectively), largely driven by socioeconomic status and social capital. Significant interactions showed that healthy lifestyles conferred stronger protection in unfavourable environments. Joint exposure to unfavourable environment and unhealthy lifestyle markedly increased mortality risk (HRs 2.5–3.5).

**Conclusions:**

Unfavourable social environments consistently predict adverse outcomes in MetS across contexts. Their synergy with lifestyle behaviours underscores the need for integrated interventions addressing both environmental and behavioural factors, especially in disadvantaged populations.

**WHAT IS ALREADY KNOWN ON THIS TOPIC**

- The prevalence of metabolic syndrome (MetS) is increasing globally and is associated with higher risks of mortality and other adverse health outcomes.
- Disadvantaged socioeconomic conditions and unhealthy lifestyles have been linked to a higher risk of MetS.
- The interaction between social-level determinants and individual-level factors in relation to health outcomes remains less well understood, and the joint association of social environment and lifestyle is still not well characterised.

**WHAT THIS STUDY ADDS**

- In this analysis of 60 423 participants from the UK Biobank and 11 170 from National Health and Nutrition Examination Survey, we found that unfavourable social environments were consistently associated with adverse health outcomes across different settings.
- Among social environment domains, socioeconomic status and social capital showed the strongest associations with all-cause mortality.
- Joint exposure to unfavourable social environment and unhealthy lifestyle was associated with higher risks of mortality and disease incidence.
- Healthy lifestyle behaviours were associated with lower risks of mortality and disease outcomes within the same social environment.

**HOW THIS STUDY MIGHT AFFECT RESEARCH, PRACTICE OR POLICY**

- Integrating both social environmental conditions and individual lifestyle factors may be important in the management of MetS.

## Introduction

 Metabolic syndrome (MetS), characterised by a constellation of metabolic abnormalities including central adiposity, hypertension, dyslipidaemia and glucose dysregulation, poses a major public health concern.^1^ Individuals with MetS face elevated risks of all-cause mortality, as well as complications including cardiovascular disease (CVD),^2^ cerebrovascular events^3^ and chronic kidney disease (CKD).^4^ Globally, the prevalence of MetS is steadily increasing, affecting approximately 37% to 42% of adults in the USA^5^ and 20%–30% in the UK.^6^ This highlights the urgent need for effective strategies to improve prognosis in affected populations.^7^

The social environment, which includes various social determinants of health related to where people live, learn, work and access healthcare, has been consistently associated with MetS.^8–11^ A comprehensive meta-analysis revealed that individuals in disadvantaged socioeconomic positions had a 1.5-fold higher risk of developing MetS compared with individuals in more advantaged positions.^12^ These social environmental factors have also been shown to play a crucial role in the prognosis and management of metabolic diseases.^13,14^ A recent study found that unemployment and food insecurity are linked to more severe disease stages of MetS.^13^ The link between a disadvantaged social environment and worsening MetS outcomes was often driven by the chronic strain of economic and social instability.^15,16^ Persistent stress kept the body’s stress-response systems activated, promoting sustained inflammation and further disrupting metabolic regulation.^17^ At the same time, structural barriers, such as living in under-resourced neighbourhoods or having limited access to high-quality healthcare, made day-to-day disease management more difficult.^18^ Financial constraints and limited health literacy further contributed to poor medication adherence and delayed care.^18,19^

However, most above studies used cross-sectional designs, which restrict their ability to establish causal inferences. Moreover, examining the impact of isolated social environmental factors on MetS outcomes may underestimate their true impact, as these factors often interact synergistically within complex social ecosystems rather than acting in isolation. For example, socioeconomic disadvantage often coincides with limited healthcare access and decreased social participation, further exacerbating health risks associated with MetS. Despite the recognised importance of social factors, there remains a gap in systematically examining the collective influence of the social environment on MetS outcomes, particularly across different healthcare systems and sociocultural contexts. Furthermore, little is known about how these broad social-level determinants interact with individual-level factors, potentially amplifying or mitigating the adverse prognosis associated with MetS.

Unlike population-level social environmental conditions, lifestyle behaviours represent another key dimension influencing MetS outcomes through individual-level pathways.^20,21^ Although prior research has independently highlighted the significance of both social environmental factors and lifestyle behaviours in MetS progression, their joint effects and potential interactions remain poorly understood. Key questions remain: do unhealthy lifestyle practices amplify the adverse consequences of an unfavourable social environment? Conversely, could a healthier lifestyle help mitigate the risks associated with social disadvantage for MetS patients? Unravelling these complex relationships is essential for designing multifaceted interventions that target both individual behaviours and broader social contexts, ultimately improving MetS outcomes more effectively.

To address these knowledge gaps, this study used nationwide cohort data from the UK and USA, which have distinct social and healthcare systems, to examine whether findings are consistent across different social security and healthcare contexts. Specifically, we aimed to: (1) examine the association between the combined social environment and clinical outcomes (including mortality, life expectancy and non-fatal events) among adults with MetS and (2) investigate the complex interplay between social environmental conditions and lifestyle behaviours in relation to health outcomes among MetS populations.

## Methods

### Study design and participants

We used two large population-based cohorts: the UK Biobank (UKB) and the US National Health and Nutrition Examination Survey (NHANES). These databases were selected to provide a comparative perspective across two distinct societal and healthcare frameworks, allowing assessment of the generalisability of our findings across different social contexts. Both cohorts included linkage to national registries and standardised recording of incident diseases and mortality using ICD-10 codes, supporting reliable outcome assessment. In addition, their data structures met the needs of this study, providing large, high-quality datasets with long-term follow-up. The UKB recruited over 500 000 adults aged 37–69 years between 2006 and 2010 across 22 centres in the UK,^22^ while NHANES has provided nationally representative samples of the US population since 1999.^23^ Further details of the two cohorts were provided in online supplemental methods.

In both UKB and NHANES, participants with MetS and aged 20 years or older at baseline were included in the analysis of mortality. MetS was defined as the presence of at least three of the following five components^24–26^: (1) abdominal obesity (elevated waist circumference: ≥102 cm in males and ≥88 cm in females); (2) elevated triglycerides (≥150 mg/dL or 1.7 mmol/L); (3) elevated blood pressure (≥130 mm Hg systolic blood pressure and/or ≥85 mm Hg diastolic blood pressure) or use of antihypertensive medication; (4) elevated fasting blood glucose (≥100 mg/dL) or glycated haemoglobin >42.0 mmol/mol or drug treatment for elevated blood glucose and (5) reduced HDL-cholesterol (High density lipoprotein cholesterol) (<40 mg/dL or 1.0 mmol/L in males; <50 mg/dL or 1.3 mmol/L in females) or use of lipid-modifying medications.

The study population excluded individuals with missing information on social environments, lifestyle factors, other covariates and follow-up data. For the analysis of incident outcomes, participants with outcomes of interest at baseline were further excluded (online supplemental figure 1).

### Outcome variables

Mortality from all causes and cause-specific (CVD, cerebrovascular disease (CBVD), CKD) and incident non-fatal outcomes (CVD, CBVD and CKD) were identified using ICD-10 codes (online supplemental table 1). In NHANES, only heart disease deaths were available for CVD mortality, whereas incident non-fatal outcomes were assessed only in the UKB. Detailed information of outcome variables is provided in online supplemental methods.

For mortality outcome, censored time was calculated as the period from the baseline year to the date of death, or the end of follow-up, whichever occurred first. For incident non-fatal outcomes, censored time was calculated as the period from the baseline year to the date of diagnosis, date of death or the end of follow-up, whichever occurred first.

### Assessment of social environments

According to Healthy People 2030 objectives, social environment related to health was classified into five domains, including economic stability (eg, employment status, poverty), education access and quality (eg, child education, language and literacy), healthcare access and quality (eg, health insurance coverage, primary care), neighbourhood and built environment (eg, natural environment, crime) and social and community context (eg, social activity, discrimination). By integrating expert knowledge and reviewing previous studies from these two studies,^27–30^ we identified four key domains of the social environment associated with MetS: socioeconomic status, social capital, healthcare access and neighbourhood and built environment. These domains encompass a range of social environment factors known to influence MetS. Due to differences in study designs and populations, the UK Biobank and NHANES studies collected different social environment variables. However, all variables were selected based on prior research using these cohorts. More details on the assessment of social environments in both cohorts were provided in online supplemental methods.

We developed a weighted composite social environment score to capture the varying impacts of social factors on health outcomes, following established epidemiological methods.^31^ The score was derived by (1) dichotomising social variables^29,32^ (online supplemental table 2), (2) assigning regression-based weights and (3) calculating a normalised weighted sum. The score was calculated by assigning regression-based weights to dichotomised social variables using the following formula: $Score=n\times \sum \left(weight_{i}\times x_{i}\right)/\sum weight$, where weight represented the regression coefficient for each factor, x was the dichotomised value of each factor, and n was the number of social environment factors. The weights for each variable were provided in online supplemental table 3. Detailed procedures are described in online supplemental methods. As a sensitivity, we also conducted an unweighted score by assigning 0 points for the advantaged level of each social environment and 1 point for the disadvantaged levels of each social environment (described in Statistical analysis). The weighted and unweighted scores ranged from 0 to 14 in UKB and from 0 to 9 in the NHANES, with higher scores indicating a less favourable social environment. Participants were categorised into tertiles, representing favourable (bottom), medium (middle) and unfavourable (top) social environment groups, to ensure balanced group sizes and enable comparison across exposure levels.

### Assessments of healthy lifestyle

In this study, four lifestyle factors, including cigarette smoking, alcohol consumption, physical activity and diet, were included based on previous evidence linking them to MetS prognosis.^33,34^ For each lifestyle factor, we assigned 1 point for a healthy level and 0 points for an unhealthy level. More information about lifestyle factors in both cohorts is provided in the online supplemental methods.

Using the same methodology as for the social environment score, we constructed a weighted healthy lifestyle score,^35^ with two key distinctions: (1) lifestyle factors were dichotomised into healthy (1 point) versus unhealthy (0 point) categories and (2) social environments, rather than lifestyle factors, were included as covariates in the adjustment models. The weights for each variable are shown in online supplemental table 4. Higher scores represented more favourable lifestyle patterns.

Participants were categorised into tertiles, representing healthy (top), medium (middle) and unhealthy (bottom) lifestyle groups. Detailed information about lifestyle factor categorisation is provided in the online supplemental methods.

### Assessment of covariates

Based on evidence from previous literature,^36–42^ we developed a directed acyclic graph to describe the potential pathways linking the social environment to MetS prognosis and adverse health outcomes (online supplemental figure 2). Guided by this framework, we selected covariates and classified them according to their roles in the pathway, distinguishing basic confounders from variables that may act as mediators.

The covariates included age, sex, lifestyle behaviours, body mass index (BMI), number of MetS components (3, 4 or 5), and baseline comorbidities. Comorbidities, including diabetes, hypertension, CVD, CBVD, CKD and cancer (yes or no), were defined by self-reported physician diagnoses (both cohorts) and disease registry (UKB only). As CBVD was not assessed in the NHANES cohort, stroke was used as a proxy, as it was the main acute form of CBVD. Details of code used to identify baseline comorbidities in both cohorts are documented in the online supplemental table 5.

Based on the hypothesised relationships among the social environment, covariates and health outcomes, we applied a stepwise adjustment approach to examine how the estimates changed with additional adjustment. The initial model included age and sex as basic confounders. Subsequent models further adjusted for lifestyle behaviours, BMI, number of MetS components (3, 4 or 5) and baseline comorbidities, which are likely to lie downstream of the social environment.

### Statistical analysis

#### Analysing the associations of social environment with mortality and disease incidence

Analyses were conducted separately in the two cohorts. Categorical variables are presented as proportions and continuous variables as means with SDs. Characteristics of both included and excluded participants were summarised.

Cox proportional hazards models assessed associations between social environment and mortality, with incremental covariates adjustment: Model 1 adjusted for age and sex; Model 2 further adjusted for lifestyle score and BMI; Model 3 additionally adjusted for the number of MetS components and baseline comorbidities. To account for the complex, multistage probability sampling design of NHANES, all analyses were performed using sample weights, primary sampling units and strata to ensure that the findings were nationally representative of the US population. Multicollinearity among covariates was assessed using variance inflation factors (VIFs) and no evidence of substantial collinearity was observed (all VIFs <5). For incident disease analysis in UKB, participants with pre-existing conditions were excluded. Fine and Grey’s proportional sub-hazards models accounted for competing risks in cause-specific mortality (with deaths from other causes as competing events) and incident diseases (with deaths as competing events).

Subgroup analyses were performed by age, sex, BMI group and number of MetS components. Interactions between social environment and subgroup variables were tested.

Population attributable fractions (PAFs) for each social environment domain were estimated from fully adjusted Cox models, with principal component analysis used to derive domain weights and account for interdependence to calculate weighted PAFs (combined and domain-specific). We assessed multicollinearity among social environment factors and found no evidence of substantial collinearity (all VIFs <5).

#### Analysing the association between social environment and life expectancy

Life expectancy was estimated using a parametric Gompertz model with age as the time scale (using ‘flexsurv’ package in R).^43^ Years of life lost were derived by comparing life expectancy between social environment groups.

#### Analysing the interaction and joint effects of lifestyle and social environment on mortality and disease incidence

We further conducted a stratified analysis by class of social environment to examine the associations between lifestyle and the risks of mortality and disease incidences among adults in different social environments.

To quantify the additive and multiplicative interactions, we additionally included a product term of social environment and lifestyle in the model. Multiplicative interactions were assessed via product terms, and additive interactions using relative excess risk due to interaction with 95% CIs. In addition, within each social environment, we estimated the PAF for each lifestyle factor to assess their relative contributions to disease burden and to identify the most influential modifiable behaviours across different social contexts.

To assess the joint associations, we further classified participants into nine groups according to social environment and lifestyle (both in tertiles). HRs were estimated relative to the group with the most favourable social environment and healthiest lifestyle.

### Sensitivity analyses

Several sensitivity analyses were conducted. First, we excluded events that occurred within the first 2 years of follow-up to reduce potential reverse causation. Second, we excluded individuals with prevalent diabetes, CVD, CBVD, CKD and cancer, as both social environment and lifestyles could be influenced by major chronic diseases. However, in this analysis, we did not exclude individuals with hypertension, given its high prevalence in the MetS population. Excluding these participants would have removed a large proportion of the sample and reduced the representativeness of the study population. Third, we used multiple imputations by chained equations method (‘mice’ package in R software)^44^ to impute all missing independent variables, testing the influence of missing variables. Fourth, to ensure the robustness of our findings to the weighting methodology, we constructed an unweighted social environment score by summing the number of unfavourable social environmental factors. This score ranged from 0 to 14 in the UK Biobank and from 0 to 9 in NHANES. Fifth, an unweighted healthy lifestyle score was constructed to account for the varying magnitudes of associations between different lifestyle factors and health outcomes. Sixth, we restricted the analysis to participants aged 40 years or older in NHANES to align with the age distribution in UKB and to reduce the concern about the potential changeability of social environment and lower lifestyle-related mortality risks in younger adults. As only one participant in UKB was aged less than 40 years, this sensitivity analysis was not performed in UKB.

All analyses were performed using SAS V.9.4 and R V.4.3.2, following Strengthening the Reporting of Observational Studies in Epidemiology (STROBE) reporting guidelines (online supplemental methods). We considered two-sided p<0.05 to be statistically significant.

## Results

### Population characteristics

In UKB, we identified 96 989 participants with MetS, and of these, we excluded 36 566 participants; In NHANES, we identified 22 954 adults aged 20 years or older with MetS, and of these, we excluded 11 784 participants (online supplemental figure 1). A total of 60 423 participants from the UKB (mean age 57.71 (SD 7.69) years; 47.15% female) and 11 170 participants from NHANES (mean age 54.25 (SD 16.64) years; 57.18% female) were included in our analysis (table 1). The distributions of social environment and lifestyle factors are shown in online supplemental figure 3. In both cohorts, most participants were concentrated in the lower to middle ranges of unfavourable social environment and unhealthy lifestyle factors, with relatively few individuals at the extremes. Based on the weighted social environment and lifestyle scores, participants were classified into tertiles. Participants in the unfavourable social environment groups were more likely to be younger and female, have unhealthy lifestyles, higher BMI and a higher prevalence of morbidities. The percentage of participants with disadvantaged levels for each social environment is provided in online supplemental table 6. Participants excluded from the current analysis due to missing information were more likely to be smokers, have lower physical activity and be non-white participants (online supplemental table 7). After a follow-up of 12.77 (SD 2.08) years, 6932 study participants in UKB were documented as having died, including 2424 from CVD, 505 from CBVD and 415 from CKD. During a follow-up of 8.03 (SD 5.07) years, the NHANES documented 1651 deaths, including 453 from CVD, 81 from CBVD and 40 from CKD.

**Table 1:** Baseline characteristics of participants according to combined social environments

	UK Biobank cohort				US NHANES cohort			
	Favourable	Medium	Unfavourable	Total population	Favourable	Medium	Unfavourable	Total population
Weighted social environments score range	≤3.79	3.79–6.33	>6.33		≤1.79	1.79–4.08	>4.08	
Age, years, mean (SD)	57.72 (7.42)	57.92 (7.86)	57.50 (7.79)	57.71 (7.69)	57.29 (15.08)	55.07 (17.59)	50.16 (16.51)	54.25 (16.64)
Sex, n (%)								
Female	8310 (41.20)	9758 (48.51)	10 424 (51.77)	28 492 (47.15)	1926 (47.89)	2072 (59.68)	2389 (64.99)	6387 (57.18)
Male	11 861 (58.80)	10 357 (51.49)	9713 (48.23)	31 931 (52.85)	2096 (52.11)	1400 (40.32)	1287 (35.01)	4783 (42.82)
Race, n (%)								
White	19 810 (98.21)	19 425 (96.57)	18 464 (91.69)	57 699 (95.49)	2528 (62.85)	1747 (50.32)	1421 (38.66)	5696 (50.99)
Black	28 (0.14)	94 (0.47)	621 (3.08)	743 (1.23)	630 (15.66)	700 (20.16)	922 (25.08)	2252 (20.16)
Other	333 (1.65)	596 (2.96)	1052 (5.22)	1981 (3.28)	864 (21.48)	1025 (29.52)	1333 (36.26)	3222 (28.85)
Body mass index, kg/m^2^, mean (SD)	30.94 (4.27)	31.36 (4.66)	32.15 (5.24)	31.48 (4.77)	32.12 (5.89)	32.87 (6.62)	33.61 (7.40)	32.68 (6.69)
Smoking status, n (%)								
Never	10 736 (53.22)	9907 (49.25)	8854 (43.97)	29 497 (48.82)	2244 (55.79)	1878 (54.09)	1729 (47.03)	5851 (52.38)
Past	8025 (39.78)	8196 (40.75)	7766 (38.57)	23 987 (39.70)	1368 (34.01)	1023 (29.46)	860 (23.39)	3251 (29.10)
Current	1410 (6.99)	2012 (10.00)	3517 (17.47)	6939 (11.48)	410 (10.19)	571 (16.45)	1087 (29.57)	2068 (18.51)
Drinking, n (%)								
≤2 drinks/week	9443 (46.81)	7528 (37.42)	5327 (26.45)	22 298 (36.90)	2138 (53.16)	2122 (61.12)	2393 (65.1)	6653 (59.56)
>2 drinks/week	10 728 (53.19)	12 587 (62.58)	14 810 (73.55)	38 125 (63.10)	1884 (46.84)	1350 (38.88)	1283 (34.9)	4517 (40.44)
Physical activity, n (%)								
Inactive	11 062 (54.84)	10 901 (54.19)	11 649 (57.85)	33 612 (55.63)	2167 (53.88)	2118 (61.00)	2539 (69.07)	6824 (61.09)
Insufficiently active	3596 (17.83)	3381 (16.81)	2942 (14.61)	9919 (16.42)	1039 (25.83)	774 (22.29)	661 (17.98)	2474 (22.15)
Active	5513 (27.33)	5833 (29.00)	5546 (27.54)	16 892 (27.96)	816 (20.29)	580 (16.71)	476 (12.95)	1872 (16.76)
Diet, n (%)								
Unhealthy	11 715 (58.08)	11 903 (59.17)	12 362 (61.39)	35 980 (59.55)	2180 (54.20)	2084 (60.02)	2438 (66.32)	6702 (60.00)
Healthy	8456 (41.92)	8212 (40.83)	7775 (38.61)	24 443 (40.45)	1842 (45.80)	1388 (39.98)	1238 (33.68)	4468 (40.00)
CVD, n (%)								
No	17 891 (88.70)	17 521 (87.10)	16 849 (83.67)	52 261 (86.49)	3506 (87.17)	2955 (85.11)	3042 (82.75)	9503 (85.08)
Yes	2280 (11.30)	2594 (12.90)	3288 (16.33)	8162 (13.51)	516 (12.83)	517 (14.89)	634 (17.25)	1667 (14.92)
Cancer, n (%)								
No	18 751 (92.96)	18 688 (92.91)	18 875 (93.73)	56 314 (93.20)	3390 (84.29)	3052 (87.9)	3337 (90.78)	9779 (87.55)
Yes	1420 (7.04)	1427 (7.09)	1262 (6.27)	4109 (6.80)	632 (15.71)	420 (12.1)	339 (9.22)	1391 (12.45)
CBVD, n (%)								
No	19 928 (98.80)	19 741 (98.14)	19 522 (96.95)	59 191 (97.96)	3887 (96.64)	3298 (94.99)	3430 (93.31)	10 615 (95.03)
Yes	243 (1.20)	374 (1.86)	615 (3.05)	1232 (2.04)	135 (3.36)	174 (5.01)	246 (6.69)	555 (4.97)
CKD, n (%)								
No	19 842 (98.37)	19 671 (97.79)	19 616 (97.41)	59 129 (97.86)	3198 (79.51)	2641 (76.07)	2808 (76.39)	8647 (77.41)
Yes	329 (1.63)	444 (2.21)	521 (2.59)	1294 (2.14)	824 (20.49)	831 (23.93)	868 (23.61)	2523 (22.59)
Diabetes, n (%)								
No	17 836 (88.42)	17 375 (86.38)	16 427 (81.58)	51 638 (85.46)	3309 (82.27)	2756 (79.38)	2835 (77.12)	8900 (79.68)
Yes	2335 (11.58)	2740 (13.62)	3710 (18.42)	8785 (14.54)	713 (17.73)	716 (20.62)	841 (22.88)	2270 (20.32)
Hypertension, n (%)								
No	4797 (23.78)	4679 (23.26)	4561 (22.65)	14 037 (23.23)	1232 (30.63)	1135 (32.69)	1340 (36.45)	3707 (33.19)
Yes	15 374 (76.22)	15 436 (76.74)	15 576 (77.35)	46 386 (76.77)	2790 (69.37)	2337 (67.31)	2336 (63.55)	7463 (66.81)
Metabolic syndrome, n (%)								
3 MetS component traits	14 293 (70.86)	13 467 (66.95)	12 533 (62.24)	40 293 (66.68)	2787 (69.29)	2265 (65.24)	2387 (64.93)	7439 (66.60)
4 MetS component traits	5075 (25.16)	5516 (27.42)	6059 (30.09)	16 650 (27.56)	1072 (26.65)	1087 (31.31)	1138 (30.96)	3297 (29.52)
5 MetS component traits	803 (3.98)	1132 (5.63)	1545 (7.70)	3480 (5.76)	163 (4.05)	120 (3.46)	151 (4.11)	434 (3.89)

CBVD, cerebrovascular disease; CKD, chronic kidney disease; CVD, cardiovascular disease; MetS, metabolic syndrome; NHANES, National Health and Nutrition Examination Survey; SD, Standard deviation.

### Association between social environment and mortality

As additional covariates were included in the models, the HRs were gradually reduced from model 1 to model 3 in both cohorts, while the associations between social environment and all-cause mortality remained statistically significant. In the UK Biobank cohort, the HR for all-cause mortality in the medium social environment group decreased from 1.19 (95% CI 1.12 to 1.27) in the initial model to 1.14 (95% CI 1.07 to 1.22) in the fully adjusted model, and from 1.91 (95% CI 1.80 to 2.02) to 1.72 (95% CI 1.62 to 1.83) in the unfavourable group (table 2). A similar pattern was observed in the NHANES cohort (table 2), where the HR decreased from 1.54 (95% CI 1.36 to 1.75) to 1.46 (95% CI 1.29 to 1.65) in the medium group and from 2.67 (95% CI 2.29 to 3.12) to 2.30 (95% CI 1.95 to 2.71) in the unfavourable group. With more unfavourable levels of social environment, participants had a progressively and significantly higher risk of all-cause mortality (P_trend_<0.001) in both cohorts.

**Table 2:** Association between the combined social environments with all-cause and cause-specific mortality among adults with MetS in the UK Biobank and US NHANES cohorts

	UK Biobank cohort				US NHANES cohort			
	Favourable	Medium	Unfavourable	P_trend_	Favourable	Medium	Unfavourable	P_trend_
Weighted social environments score range	≤3.79	3.79–6.33	>6.33		≤1.79	1.79–4.08	>4.08	
All-cause mortality								
Number of participants	20 171	20 115	20 137		4022	3472	3676	
Number of cases	1803	2096	3033		491	678	482	
HR (95% CI) in model 1	1.00 (ref.)	1.19 (1.12 to 1.27)	1.91 (1.80 to 2.02)	<0.001	1.00 (ref.)	1.54 (1.36 to 1.75)	2.67 (2.29 to 3.12)	<0.001
HR (95% CI) in model 2	1.00 (ref.)	1.18 (1.11 to 1.26)	1.85 (1.74 to 1.96)	<0.001	1.00 (ref.)	1.52 (1.34 to 1.73)	2.59 (2.22 to 3.03)	<0.001
HR (95% CI) in model 3	1.00 (ref.)	1.14 (1.07 to 1.22)	1.72 (1.62 to 1.83)	<0.001	1.00 (ref.)	1.46 (1.29 to 1.65)	2.30 (1.95 to 2.71)	<0.001
CVD mortality								
Number of cases	545	703	1176		155	169	129	
HR (95% CI) in model 1	1.00 (ref.)	1.35 (1.20 to 1.51)	2.48 (2.24 to 2.74)	0.005	1.00 (ref.)	1.33 (1.07 to 1.66)	1.93 (1.51 to 2.46)	<0.001
HR (95% CI) in model 2	1.00 (ref.)	1.32 (1.18 to 1.47)	2.29 (2.06 to 2.53)	<0.001	1.00 (ref.)	1.31 (1.05 to 1.63)	1.87 (1.47 to 2.39)	<0.001
HR (95% CI) in model 3	1.00 (ref.)	1.23 (1.10 to 1.37)	1.93 (1.74 to 2.14)	<0.001	1.00 (ref.)	1.19 (0.95 to 1.49)	1.58 (1.23 to 2.03)	<0.001
CBVD mortality								
Number of cases	117	159	229		29	34	18	
HR (95% CI) in model 1	1.00 (ref.)	1.32 (1.04 to 1.68)	2.04 (1.63 to 2.55)	0.170	1.00 (ref.)	1.66 (0.98 to 2.81)	1.24 (0.63 to 2.43)	0.270
HR (95% CI) in model 2	1.00 (ref.)	1.31 (1.03 to 1.66)	1.98 (1.58 to 2.49)	0.027	1.00 (ref.)	1.69 (1.00 to 2.87)	1.28 (0.65 to 2.51)	0.230
HR (95% CI) in model 3	1.00 (ref.)	1.22 (0.96 to 1.55)	1.67 (1.34 to 2.10)	<0.001	1.00 (ref.)	1.65 (0.97to 2.81)	1.17 (0.58 to 2.36)	0.390
CKD mortality								
Number of cases	77	123	215		7	21	12	
HR (95% CI) in model 1	1.00 (ref.)	1.69 (1.27 to 2.25)	3.06 (2.35 to 3.98)	0.092	1.00 (ref.)	6.78 (2.32 to 19.83)	5.17 (1.59 to 16.83)	<0.001
HR (95% CI) in model 2	1.00 (ref.)	1.64 (1.23 to 2.18)	2.73 (2.09 to 3.56)	0.001	1.00 (ref.)	6.67 (2.30 to 19.35)	5.03 (1.57 to 16.15)	<0.001
HR (95% CI) in model 3	1.00 (ref.)	1.42 (1.07 to 1.90)	2.02 (1.54 to 2.66)	<0.001	1.00 (ref.)	5.55 (1.88 to 16.38)	3.39 (1.04 to 11.06)	0.006

Model 1 adjusted for age and sex. Model 2 additionally adjusted for healthy lifestyle score, body mass index; Model 3 additionally adjusted for the number of metabolic component traits and prevalence of CVD, CBVD, CKD, diabetes, cancer and hypertension.

CBVD, cerebrovascular disease; CI, Confidence interval; CKD, chronic kidney disease; CVD, cardiovascular disease; HR, hazard ratio; MetS, metabolic syndrome; NHANES, National Health and Nutrition Examination Survey.

Compared with the favourable social environment group, a consistent pattern of elevated mortality risks was observed across multiple diseases, though with varying magnitudes between the UKB and NHANES cohorts (table 2). In the fully adjusted model, CVD mortality demonstrated increased risks in both cohorts, with UKB showing a significant trend (medium: HR 1.23 (95% CI 1.10 to 1.37); unfavourable: HR 1.93 (95% CI 1.74 to 2.14); P_trend_<0.001) and NHANES showing notably higher risks, particularly in the unfavourable group (HR 1.58 (95% CI 1.23 to 2.03); P_trend_<0.001). For CBVD mortality, only the UKB cohort showed significant trends of increased risk (medium: HR 1.22 (95% CI 0.96 to 1.55); unfavourable: HR 1.67 (1.34 to 2.10); P_trend_<0.001). For CKD mortality, both cohorts showed significant trends of increased risk: UKB (medium: HR 1.42 (95% CI 1.07 to 1.90); unfavourable: HR 2.02 (95% CI 1.54 to 2.66); P_trend_<0.001) and NHANES (medium: HR 5.55 (95% CI 1.88 to 16.38); unfavourable: HR 3.39 (95% CI 1.04 to 11.06); P_trend_=0.006). Across models, the HRs in the UK Biobank cohort showed a consistent reduction from model 1 to model 3 for all outcomes. A similar attenuation pattern was observed for most outcomes in the NHANES cohort, while for CBVD mortality, the HR increased slightly from model 1 to model 2 and then decreased in model 3.

Social environment contributed substantially to mortality, accounting for 17.0% and 24.1% of all-cause mortality in UKB and NHANES, respectively. This contribution was primarily driven by socioeconomic status (7.2%) and social capital (5.5%) in UKB, while the primary contributors were social capital (9.8%) and healthcare (8.0%) in NHANES. For cause-specific mortality, the highest attributable fractions were observed for CVD mortality in UKB (44.2%) and CKD mortality in NHANES (44.1%). Socioeconomic status showed stronger effects in UKB (11.4%–24.3%), while social capital demonstrated consistent contributions across both cohorts (UKB: 14.4%–18.3%; NHANES: 0.7%–20.7%). Healthcare access was particularly influential in NHANES, especially for CKD mortality (17.2%) (online supplemental table 8).

Subgroup analyses for the associations between social environment and all-cause and cause-specific mortality are presented in online supplemental figure 4. The association between social environment and both all-cause and cause-specific mortality was significantly stronger in younger adults (<60 years) compared with older adults (≥60 years), demonstrating a significant age interaction effect. Results from sensitivity analyses were largely consistent with the main analysis (online supplemental tables 9 and 10).

### Association between social environment and life expectancy

At age 45, compared with those with a favourable social environment, the mean life expectancy of participants in the medium and unfavourable social environment groups was shortened by 0.65 years (95% CI 0.57 to 0.74) and 3.49 years (3.40 to 3.57) in UKB, and by 3.79 years (3.53 to 4.05) and 7.81 years (7.56 to 8.07) in NHANES, respectively (online supplemental table 11). Estimates of life expectancy at the ages of 45, 55, 65 and 75 years can be seen in online supplemental table 12.

### Association between social environment and future disease risk

In the UKB cohort, 12 626 participants had incident CVD over a mean follow-up of 11.7 (SD 3.3) years, 3541 participants had incident CBVD over 12.6 (SD 2.4) years, and 5284 participants had incident CKD over 12.4 (SD 2.6) years. In the adjusted model, compared with the favourable social environmental group, the HR for CVD was 1.08 (95% CI 1.03 to 1.13) in the medium social environmental group and 1.28 (95% CI 1.23 to 1.34) in the unfavourable group, respectively. For CBVD, the HR was 1.15 (95% CI 1.06 to 1.26) in the medium group and 1.46 (95% CI 1.35 to 1.59) in the unfavourable group, respectively. For CKD, the HR was 1.08 (95% CI 1.00 to 1.16) in the medium group and 1.32 (95% CI 1.24 to 1.42) in the unfavourable group, respectively (table 3). Significant positive gradients of associations were observed between more unfavourable social environment group and the risk of incident CVD, CBVD and CKD (all P_trend_<0.001) (table 3).

**Table 3:** Association between the combined social environments and diseases incidence among adults with MetS in the UK Biobank

	Favourable	Medium	Unfavourable	P_trend_
Weighted social environments score range	≤3.79	3.79–6.33	>6.33	
CVD incidence				
Number of participants	17 860	17 550	16 829	
Number of cases	3952	4134	4540	
HR (95% CI) in model 1	1.00 (ref.)	1.10 (1.06 to 1.15)	1.37 (1.31 to 1.43)	<0.001
HR (95% CI) in model 2	1.00 (ref.)	1.09 (1.05 to 1.14)	1.31 (1.26 to 1.37)	<0.001
HR (95% CI) in model 3	1.00 (ref.)	1.08 (1.03 to 1.13)	1.28 (1.23 to 1.34)	<0.001
CBVD incidence				
Number of participants	19 810	19 686	19 582	
Number of cases	981	1145	1415	
HR (95% CI) in model 1	1.00 (ref.)	1.19 (1.09 to 1.29)	1.59 (1.46 to 1.72)	<0.001
HR (95% CI) in model 2	1.00 (ref.)	1.18 (1.09 to 1.29)	1.56 (1.44 to 1.70)	<0.001
HR (95% CI) in model 3	1.00 (ref.)	1.15 (1.06 to 1.26)	1.46 (1.35 to 1.59)	<0.001
CKD incidence				
Number of participants	19 839	19 694	19 416	
Number of cases	1462	1684	2138	
HR (95% CI) in model 1	1.00 (ref.)	1.14 (1.07 to 1.23)	1.57 (1.47 to 1.68)	<0.001
HR (95% CI) in model 2	1.00 (ref.)	1.12 (1.05 to 1.20)	1.48 (1.38 to 1.58)	<0.001
HR (95% CI) in model 3	1.00 (ref.)	1.08 (1.00 to 1.16)	1.32 (1.24 to 1.42)	<0.001

Only those free from the corresponding disease at baseline were included in the analysis for diseases incidence. Model 1 adjusted for age and sex. Model 2 additionally adjusted for healthy lifestyle score, body mass index. Model 3 additionally adjusted for the number of metabolic component traits and prevalence of CVD, CBVD, CKD, diabetes, cancer and hypertension. Prevalence of corresponding disease was not included in model 3 for the analysis of incident diseases.

CBVD, cerebrovascular disease; CI, Confidence interval; CKD, chronic kidney disease; CVD, cardiovascular disease; HR, hazard ratio; MetS, metabolic syndrome.

The results remained largely consistent across different subgroups (online supplemental figure 5). In sensitivity analyses, the result remained largely consistent with the main analyses (online supplemental table 13).

### Interaction and joint effect of lifestyle and social environment on mortality and disease incidence

**Figure 1:**
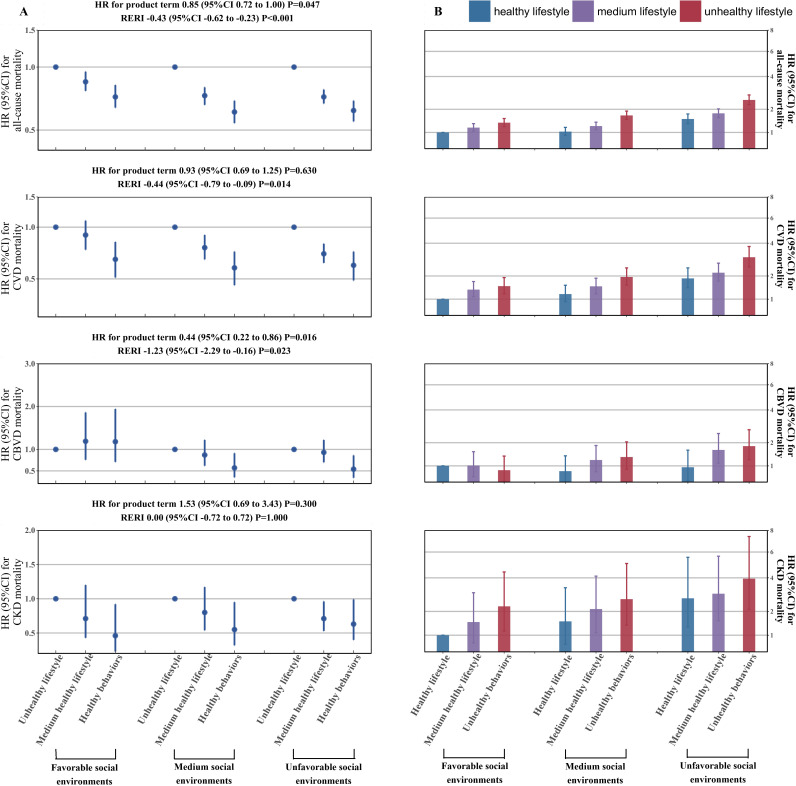
The association of social environments, lifestyle and all-cause and cause-specific mortality among adults with MetS in UK Biobank cohort. (A) The association of lifestyle with all-cause and cause-specific mortality by social environments; (B) The joint effect of social environments and lifestyle on all-cause and cause-specific mortality. Participants were grouped into nine groups according to social environments (unfavourable, medium and favourable) and healthy lifestyle score (unhealthy, medium and healthy) with those with favourable social environments and healthy lifestyle as the reference group. HRs were adjusted for age, sex, body mass index, the number of metabolic component traits and prevalence of CVD, CBVD, CKD, diabetes, cancer and hypertension. Multiplicative interaction was evaluated using HRs for the product term between the healthy lifestyle score (unhealthy lifestyle vs healthy lifestyle) and social environments (unfavourable vs favourable), and the multiplicative interaction was statistically significant when its CI did not include 1. Additive interaction was evaluated using relative excess risk due to interaction (RERI) between the healthy lifestyle score (unhealthy lifestyle vs healthy lifestyle) and social environments (unfavourable vs favourable) and the additive interaction was statistically significant when its CI did not include 0. CBVD, cerebrovascular disease; CKD, chronic kidney disease; CVD, cardiovascular disease; MetS, metabolic syndrome; HR, hazard ratio; CI, Confidence interval.

**Figure 2:**
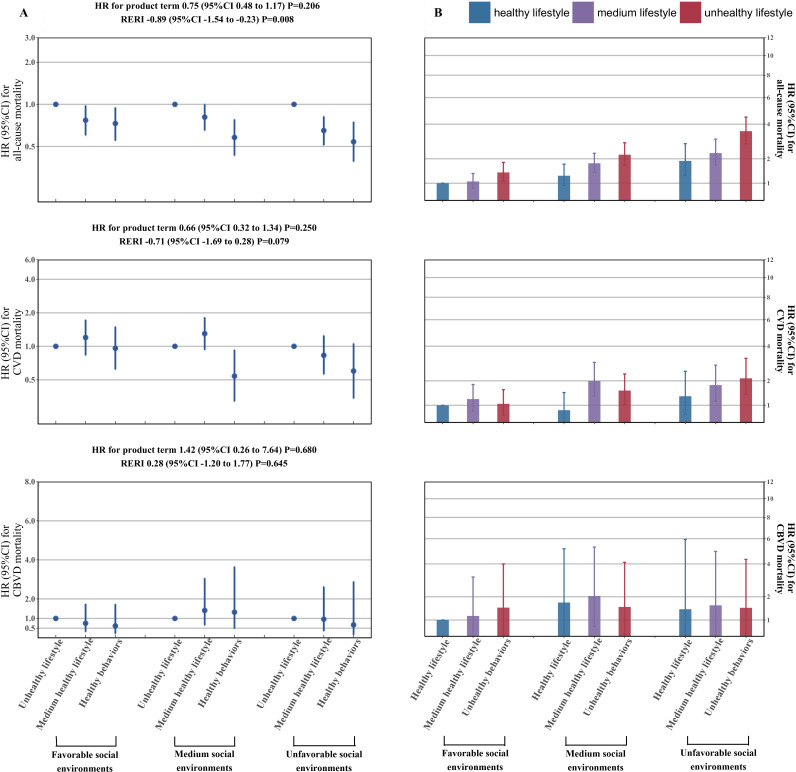
The association of social environments, lifestyle and all-cause and cause-specific mortality among adults with MetS in US NHANES cohort. (A) The association of lifestyle with all-cause and cause-specific mortality by social environments; (B) The joint effect of social environments and lifestyle on all-cause and cause-specific mortality. Participants were grouped into nine groups according to social environments (unfavourable, medium and favourable) and healthy lifestyle score (unhealthy, medium and healthy) with those with favourable social environments and healthy lifestyle as the reference group. HRs were adjusted for age, sex, body mass index, the number of metabolic component traits, and prevalence of CVD, CBVD, CKD, diabetes, cancer and hypertension. Multiplicative interaction was evaluated using HRs for the product term between the healthy lifestyle score (unhealthy lifestyle vs healthy lifestyle) and social environments (unfavourable vs favourable), and the multiplicative interaction was statistically significant when its CI did not include 1. Additive interaction was evaluated using relative excess risk due to interaction (RERI) between the healthy lifestyle score (unhealthy lifestyle vs healthy lifestyle) and social environments (unfavourable vs favourable), and the additive interaction was statistically significant when its CI did not include 0. CBVD, cerebrovascular disease; CKD, chronic kidney disease; CVD, cardiovascular disease; MetS, metabolic syndrome; NHANES, National Health and Nutrition Examination Survey; HR, hazard ratio; CI, Confidence interval.

Our analyses revealed significant interactions between social environment and lifestyle factors across both cohorts. In the UKB cohort, both multiplicative and additive interactions were observed for all-cause mortality and CBVD mortality, as well as CBVD incidence (figure 1, online supplemental tables 14 and 15). The NHANES cohort demonstrated additive interactions for all-cause mortality and multiplicative interactions for CKD mortality (figure 2, online supplemental table 16).

The association between lifestyle scores and health outcomes varied across different social environment strata, with consistently stronger protective effects of healthy lifestyle observed among individuals from unfavourable social environments, particularly in NHANES. Specifically, when comparing healthy versus unhealthy lifestyle, the HRs for all-cause mortality were 0.72 (95% CI 0.64 to 0.82) in the favourable social environment group, decreasing to 0.62 (95% CI 0.55 to 0.69) in the unfavourable social environment group. Similar patterns were observed for CVD mortality in both cohorts and for CVD and CKD incidence in UKB (figure 1, online supplemental tables 14–16). These findings remained robust across multiple sensitivity analyses (online supplemental tables 17–20). Across all social environment strata, cigarette smoking accounted for the largest PAF among the four lifestyle factors. In the UK Biobank cohort, the PAF for never smoking was −17.4% in the favourable social environment group, −22.5% in the medium group and −20.6% in the unfavourable group. A similar pattern was observed in the NHANES cohort, the PAFs of never smoking were −13.7%, −13.8% and −18.2% in the favourable, medium and unfavourable social environment groups, respectively (online supplemental table 21).

Joint exposure analyses revealed substantial combined effects of social environment and lifestyle factors on MetS outcomes. Participants with both an unfavourable social environment and an unhealthy lifestyle showed significantly higher mortality risks compared with those with a favourable social environment and a healthy lifestyle (HR for all-cause mortality: 2.50 (2.24 to 2.79) in UKB; 3.54 (2.79 to 4.49) in NHANES). This pattern was consistent across cause-specific mortality and disease incidence outcomes (figures 1 and 2, online supplemental tables 22–24), and the results remained robust in sensitivity analyses (online supplemental tables 25–28).

## Discussion

In this comprehensive analysis of two large prospective cohorts from the UK and USA, unfavourable social environments were consistently associated with adverse health outcomes in individuals with MetS, despite differences in social contexts. We found that unfavourable social environments were significantly associated with increased risks of all-cause mortality (HR 1.72–2.30), cause-specific mortality and elevated risks of incident CVD and CBVD, with a reduction in life expectancy of 3.49–7.81 years at age 45. However, in the NHANES cohort, estimates for CKD mortality showed a slightly higher HR in the medium group compared with the unfavourable group, along with wider CIs, which may be related to the limited number of CKD deaths (n=40). These findings should be interpreted with caution. Furthermore, significant interactions between social environments and lifestyle behaviours were observed, with healthy lifestyles providing stronger protection in disadvantaged settings, among which never smoking showed the strongest protective association. Joint exposure to unfavourable social environment and unhealthy lifestyle was associated with higher mortality and disease incidence risks (HR: 2.50–3.54 for all-cause mortality), compared with those with favourable social environment and healthy lifestyle, highlighting the synergistic association of these factors on adverse health outcomes.

Previous studies have established that adverse social environments increase MetS risk and exacerbate disease management. For instance, individuals with limited financial resources often delay essential medical care,^45^ accelerating progression of MetS.^46,47^ Limited access to medications and suboptimal health literacy often lead to poor treatment adherence, creating a vicious cycle where poor health further exacerbates the challenges faced by individuals in disadvantaged settings.^48,49^ However, findings across prior studies have remained inconsistent due to differences in study populations, sociocultural contexts and methodological approaches.^50^ In addition, the association between social environments and health outcomes was inherently complex and interconnected. For example, the lack of health insurance often coincides with other disadvantages, such as low income and limited access to quality healthcare.^51,52^ To better capture these combined effects, we conducted a weighted composite social environment score that integrated multiple social determinants, including socioeconomic status, social capital, healthcare accessibility and neighbourhood characteristics.^53^ This dual analytical framework allowed a more nuanced understanding of how these factors were related to health outcomes. Compared with previous studies,^54,55^ we also accounted for a wider range of potential confounders, including MetS-related comorbidities and lifestyle behaviours, using stepwise adjustment to better characterise their contributions to the observed associations. Furthermore, unlike prior small-scale (typically n<10 000) studies, our large-scale analysis offers more robust and widely applicable findings.^56,57^ This comprehensive approach offered a deeper understanding of the complex interplay between social environment and health outcomes in MetS populations.

By integrating multidomain social environments, we found that both the composite measure and individual components were significantly associated with population-level health disparities in MetS. These findings were consistent with previous research, indicating that socioeconomic status and social capital emerged as key determinants of mortality risk.^58,59^ We also observed attenuation of effect estimates after further adjustment for lifestyle factors, BMI and comorbidities, suggesting that these factors may partly explain the observed associations. In the interaction analysis, unhealthy lifestyle behaviours were consistently associated with elevated all-cause mortality risk across both cohorts, regardless of social environmental conditions, which reinforces and extends previous evidence. For instance, a meta-analysis revealed that structured lifestyle interventions effectively resolved components of MetS and reduced the severity of metabolic abnormalities.^60–62^ At the same time, the magnitude of these associations appeared greater among individuals in more disadvantaged social environments, suggesting that individuals with disadvantaged socioeconomic status were more affected by the adverse effects of unhealthy lifestyles.^63,64^

Several mechanisms might help explain these findings. First, chronic exposure to adverse social conditions, such as low socioeconomic environment or social isolation, might lead to sustained activation of stress-response systems, including the hypothalamic–pituitary–adrenal axis and the sympathetic nervous system.^17^ This dysregulation could increase cortisol levels, promoting visceral fat accumulation and impairing insulin sensitivity, thereby accelerating metabolic deterioration.^17,42^ In contrast, healthy behaviours, including regular physical activity and a balanced diet, could improve insulin sensitivity and lipid profiles, thereby slowing metabolic deterioration.^65^ Second, social disadvantage, including limited access to healthcare and neighbourhood deprivation, had been linked to higher levels of inflammatory markers such as C reactive protein and interleukin-6.^66,67^ These processes might further aggravate oxidative stress and endothelial dysfunction, increasing the risk of adverse cardiovascular outcomes.^68^ Healthy lifestyles might help mitigate these effects through anti-inflammatory and antioxidant pathways.^69^ Third, features of the built environment could influence health-related behaviours.^70,71^ Limited access to healthy foods and safe spaces for physical activity might constrain lifestyle choices and promote conditions that sustain metabolic abnormalities.^72^ Individuals who maintain healthier lifestyles might be more likely to engage in other beneficial health practices, such as better treatment adherence and proactive healthcare use, which might further improve outcomes.^73^

Our findings might help to understand the management of MetS in different social contexts. In this study, among the four lifestyle factors (cigarette smoking, alcohol consumption, physical activity and diet), non-smoking showed the largest PAF across all social environments, suggesting that smoking cessation might be an important factor in relation to mortality risk among populations with MetS. At the same time, the varying impact of lifestyle behaviours across different social contexts suggested that the lifestyle interventions may be more or less effective depending on the social environmental conditions of the target populations.^74^ These findings indicated that focusing on a single behaviour alone may not fully capture the complexity of health outcomes in MetS. Recent studies also showed that lifestyle interventions yield significantly different outcomes across socioeconomic groups,^75,76^ highlighting the need for integrated behavioural and environmental strategies targeting disadvantaged MetS populations. Other evidence also suggested that lifestyle interventions are most effective when combined with improvements in social environmental conditions and show better outcomes than single-focus approaches.^63,77–79^ This insight suggested that approaches integrating both social environmental conditions and individual lifestyle factors may be important in the management of MetS.

Several limitations should be considered. First, the construction of combined social environment scores was based on available data from the UKB and NHANES, which posed challenges in creating identical metrics across cohorts. Although variables were constructed as closely as possible, some domains of the social environment were less comprehensively captured in NHANES. As this study was based on a cross-cohort design, such differences are difficult to fully avoid and may lead to residual confounding and incomplete representation of the social environment construct. Nevertheless, the consistent associations across both cohorts suggest the broader generalisability of our findings. Second, both social environment and lifestyle data were self-reported and collected at a single time point, which could introduce potential measurement error and prevent tracking changes over time, highlighting the need for future studies with repeated assessments throughout adulthood. Third, the weights used to construct the weighted social environment score were derived from the regression coefficients of social factors in relation to all-cause mortality. As a result, the weighted score might not be fully independent of the outcome, which could introduce a degree of overfitting and influence the estimated associations. However, this weighting approach is commonly used in epidemiological studies to reflect differences in the relative importance of social environmental factors. To address this concern, we conducted sensitivity analyses using unweighted scores, which yielded consistent results and supported the robustness of the main findings. Fourth, our weighted lifestyle score may not fully capture complex interactions between lifestyle components, and its study-specific weights may limit generalisability. Fifth, both cohorts have sampling limitations: the UKB suffers from healthy volunteer bias, while NHANES excludes individuals residing in healthcare facilities, potentially underestimating associations. Sixth, while our social environment score incorporates multiple factors, it may not fully capture the complex interactions between these components. Future research would benefit from more advanced methodologies to assess the cumulative impacts of social environments. Seventh, despite controlling for major confounders, residual confounders remain possible. Additionally, lifestyle and MetS-related characteristics may mediate observed associations, necessitating careful interpretation of fully adjusted models, though the attenuated estimates remain statistically significant. Lastly, the exclusion of participants with missing covariates, who tended to have more disadvantaged social environments, may have underestimated health outcome inequities. However, sensitivity analyses using imputed data yielded consistent results, supporting the robustness of our results.

## Conclusions

In this comprehensive analysis of UKB and NHANES, we consistently found that unfavourable social environments were associated with adverse health outcomes in individuals with MetS, despite differences in social contexts, demonstrating that individuals in unfavourable social environments faced significantly higher risks of all-cause and cause-specific mortality, CVD incidence, and shorter life expectancy. While healthy lifestyle behaviours were protective across all social strata, we observed stronger beneficial effects among individuals in unfavourable social environments, suggesting that lifestyle interventions may be especially effective for disadvantaged populations. These findings highlight the necessity of integrated interventions addressing both social environmental and lifestyle behaviours. The consistency of results across two distinct societal contexts emphasises their fundamental importance and calls for systematic policy responses to address social environmental disparities in health outcomes.

## Supplementary material

10.1136/bmjph-2025-004599 online supplemental file 1

10.1136/bmjph-2025-004599 online supplemental file 2

## Data Availability

Data are available in a public, open access repository.
