# Supplementary material for: Social environments and lifestyles linked to mortality and future health risks in metabolic syndrome: evidence from UK and US nationwide cohort studies
Source: BMJ Public Health. 2026 Aug 5;4(3):e004599. doi: 10.1136/bmjph-2025-004599 (PMC13475095; doi:10.1136/bmjph-2025-004599)
Supplement: Supplementary file 1 [file bmjph-4-3-s001.pdf]

# Additional File 1

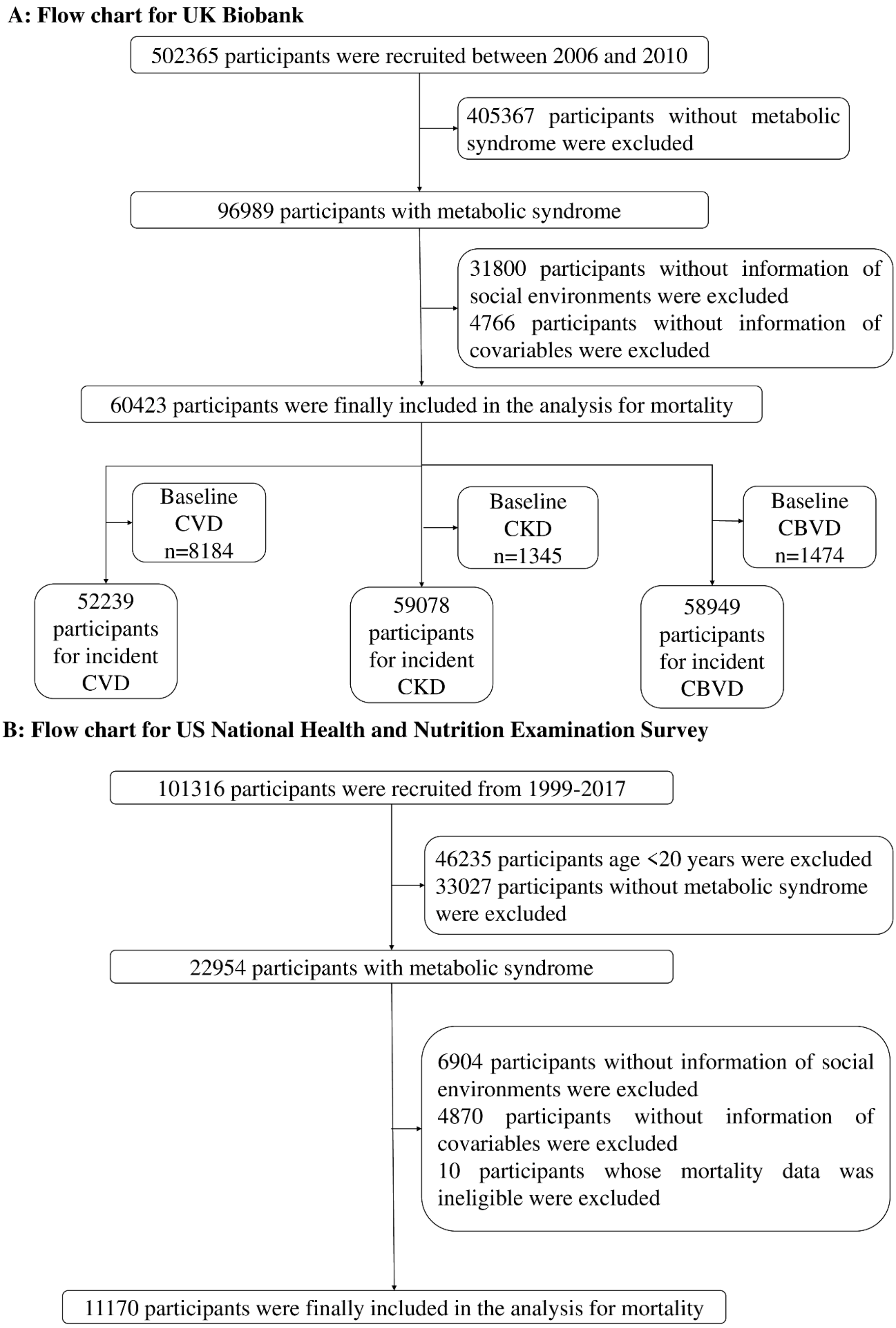


**Supplementary Figure 1. Flowchart of the study.** CVD, cardiovascular disease; CBVD, cerebrovascular disease; CKD, chronic kidney disease

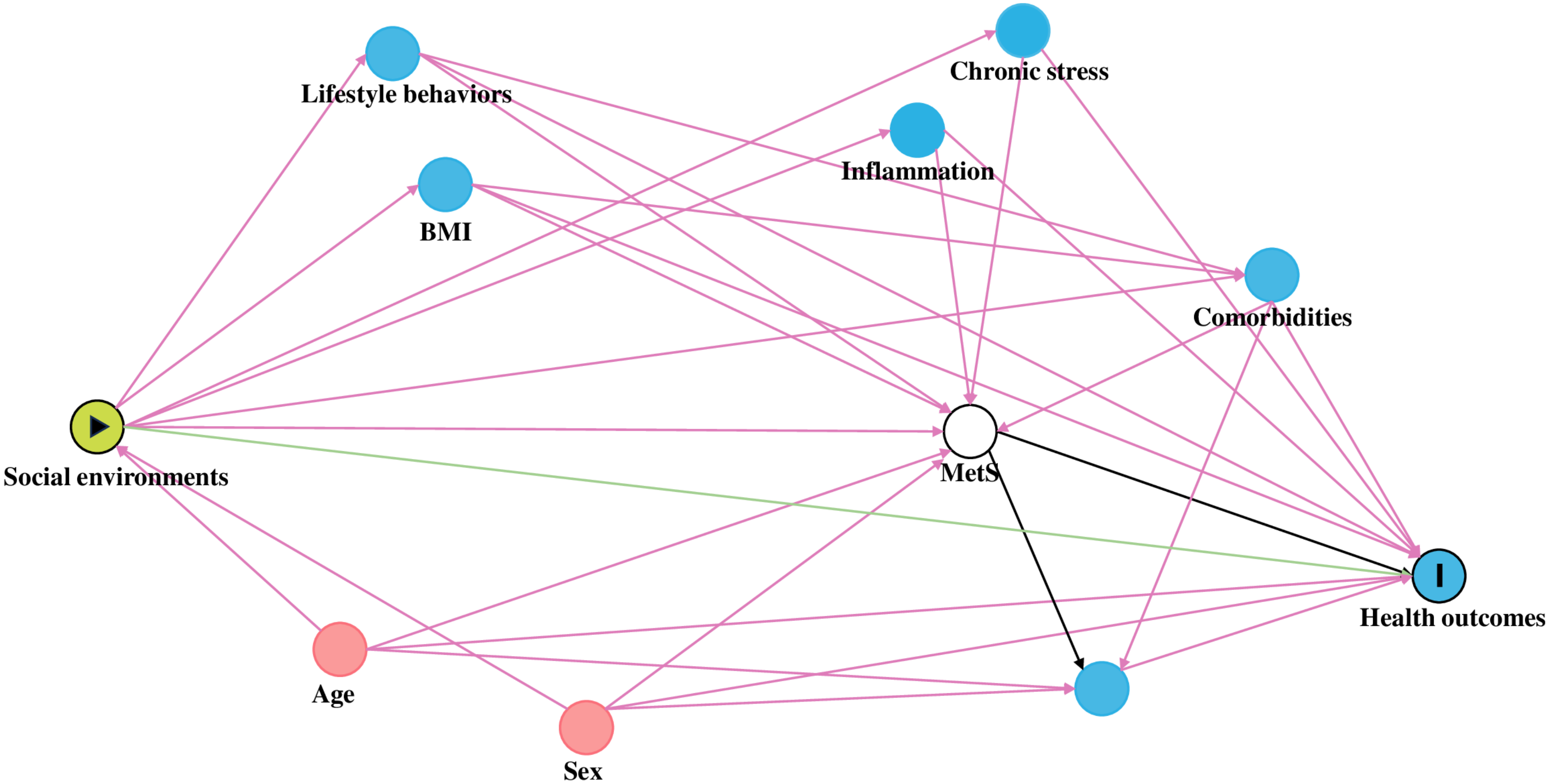


**Supplementary Figure 2 The Directed Acyclic Graph (DAG) of potential confounders**

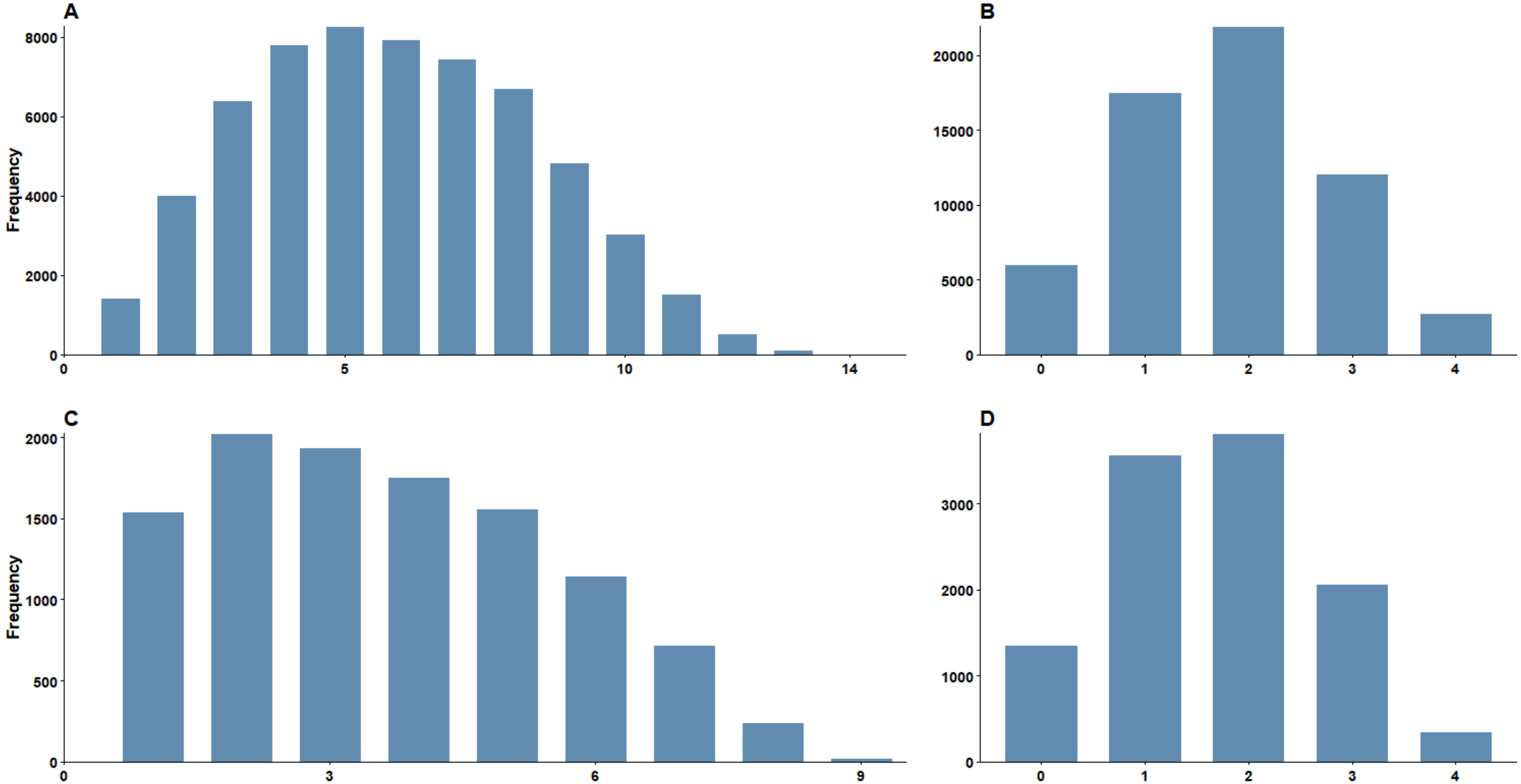


**Supplementary Figure 3 Distribution of social environment and lifestyle factors.** Panel A, the distribution of the number of unfavorable social environment factors in UK Biobank; Panel B, the distribution of the number of unhealthy lifestyle factors in UK Biobank. Panel C, the distribution of the number of unfavorable social environment factors in NHANES; Panel D, the distribution of the number of unhealthy lifestyle factors in NHANES.

| | UK Biobank | | | | | US NHANES | | | | |
|---|---|---|---|---|---|---|---|---|---|---|
| Subgroup | Case/Total | HR(95%CI) | P for trend | | P for interaction | Case/Total | HR(95%CI) | P for trend | | P for interaction |
| **All-cause mortality** | | | | | | | | | | |
| Age | | | | | 0.812 | | | | | 0.003 |
| <60 | 1837/30696 | 2.00 (1.78, 2.25) | <0.001 | | | 323/6369 | 2.63(1.82, 3.80) | <0.001 | | |
| ≥60 | 5095/29727 | 1.64 (1.53, 1.76) | <0.001 | | | 1328/4801 | 1.61(1.31, 1.97) | <0.001 | | |
| Sex | | | | | 0.991 | | | | | 0.236 |
| Female | 2471/28492 | 1.63 (1.47, 1.80) | <0.001 | | | 833/6387 | 1.75(1.37, 2.23) | <0.001 | | |
| Male | 4461/31931 | 1.79 (1.66, 1.92) | <0.001 | | | 818/4783 | 2.31(1.79, 2.98) | <0.001 | | |
| Body mass index, kg/m2 | | | | | 0.785 | | | | | 0.265 |
| <25 | 349/3005 | 1.99 (1.52, 2.62) | <0.001 | | | 206/844 | 1.65(1.06, 2.58) | 0.011 | | |
| ≤29.9 | 2369/21556 | 1.72 (1.56, 1.91) | <0.001 | | | 595/3443 | 1.77(1.32, 2.37) | <0.001 | | |
| >29.9 | 4214/35862 | 1.71 (1.58, 1.84) | <0.001 | | | 850/6883 | 2.18(1.74, 2.72) | <0.001 | | |
| Metabolic syndrome | | | | | 0.406 | | | | | 0.461 |
| 3 MetS component traits | 4136/40293 | 1.69 (1.57, 1.82) | <0.001 | | | 1056/7439 | 2.05(1.60, 2.62) | <0.001 | | |
| 4or5 MetS component traits | 2796/20130 | 1.80 (1.64, 1.98) | <0.001 | | | 595/3731 | 1.86(1.41, 2.46) | <0.001 | | |
| **CVD mortality** | | | | | | | | | | |
| Age | | | | | 0.001 | | | | | 0.004 |
| <60 | 607/30696 | 2.60 (2.10, 3.22) | <0.001 | | | 323/6369 | 2.82 (1.45, 5.49) | 0.002 | | |
| ≥60 | 1817/29727 | 1.81 (1.61, 2.04) | <0.001 | | | 1328/4801 | 1.15 (0.86, 1.54) | 0.220 | | |
| Sex | | | | | 0.620 | | | | | 0.740 |
| Female | 650/28492 | 1.87 (1.52, 2.31) | <0.001 | | | 833/6387 | 1.43 (0.98, 2.08) | 0.064 | | |
| Male | 1774/31931 | 2.03 (1.80, 2.29) | <0.001 | | | 818/4783 | 1.35 (0.95, 1.92) | 0.074 | | |
| Body mass index, kg/m2 | | | | | 0.670 | | | | | 0.630 |
| <25 | 92/3005 | 1.59 (0.94, 2.67) | 0.079 | | | 206/844 | 1.27 (0.67, 2.42) | 0.450 | | |
| ≤29.9 | 740/21556 | 2.20 (1.82, 2.65) | <0.001 | | | 595/3443 | 1.22 (0.76, 1.95) | 0.280 | | |
| >29.9 | 1592/35862 | 1.90 (1.67, 2.16) | <0.001 | | | 850/6883 | 1.51 (1.07, 2.12) | 0.017 | | |
| Metabolic syndrome | | | | | 0.850 | | | | | 0.880 |
| 3 MetS component traits | 1348/40293 | 2.02 (1.76, 2.31) | <0.001 | | | 1056/7439 | 1.43 (1.03, 1.98) | 0.021 | | |
| 4or5 MetS component traits | 1076/20130 | 1.94 (1.65, 2.27) | <0.001 | | | 595/3731 | 1.25 (0.85, 1.85) | 0.220 | | |
| **CBVD mortality** | | | | | | | | | | |
| Age | | | | | 0.010 | | | | | 0.190 |
| <60 | 93/30696 | 2.95 (1.67, 5.22) | <0.001 | | | 323/6369 | 5.22 (0.66, 41.16) | 0.075 | | |
| ≥60 | 412/29727 | 1.49 (1.17, 1.92) | 0.001 | | | 1328/4801 | 0.81 (0.37, 1.78) | 0.990 | | |
| Sex | | | | | 0.310 | | | | | 0.690 |
| Female | 196/28492 | 1.43 (0.99, 2.07) | 0.051 | | | 833/6387 | 0.90 (0.38, 2.17) | 0.920 | | |
| Male | 309/31931 | 1.89 (1.42, 2.50) | <0.001 | | | 818/4783 | 1.17 (0.38, 3.60) | 0.350 | | |
| Body mass index, kg/m2 | | | | | 0.026 | | | | | 0.460 |
| <25 | 33/3005 | 8.19 (1.90, 35.36) | <0.001 | | | 206/844 | 1.83 (0.31, 10.80) | 0.330 | | |
| ≤29.9 | 187/21556 | 1.59 (1.10, 2.29) | 0.013 | | | 595/3443 | 1.03 (0.34, 3.13) | 0.920 | | |
| >29.9 | 285/35862 | 1.56 (1.16, 2.09) | 0.002 | | | 850/6883 | 0.80 (0.27, 2.35) | 0.960 | | |
| Metabolic syndrome | | | | | 0.680 | | | | | 0.330 |
| 3 MetS component traits | 285/40293 | 1.62 (1.21, 2.16) | 0.001 | | | 1056/7439 | 1.13 (0.51, 2.49) | 0.600 | | |
| 4or5 MetS component traits | 220/20130 | 1.84 (1.28, 2.63) | 0.001 | | | 595/3731 | 0.56 (0.11, 2.76) | 0.920 | | |
| **CKD mortality** | | | | | | | | | | |
| Age | | | | | 0.003 | | | | | 0.560 |
| <60 | 74/30696 | 5.63 (2.15, 14.75) | <0.001 | | | 323/6369 | 1.91 (0.13, 28.66) | 0.670 | | |
| ≥60 | 341/29727 | 1.77 (1.33, 2.37) | <0.001 | | | 1328/4801 | 3.67 (0.95, 14.11) | 0.007 | | |
| Sex | | | | | 0.610 | | | | | 0.750 |
| Female | 124/28492 | 2.08 (1.24, 3.48) | 0.001 | | | 833/6387 | 2.37 (0.46, 12.34) | 0.340 | | |
| Male | 291/31931 | 2.11 (1.53, 2.90) | <0.001 | | | 818/4783 | 2.34 (0.34, 16.20) | 0.007 | | |
| Body mass index, kg/m2 | | | | | 0.620 | | | | | 0.120 |
| <25 | 23/3005 | 1.44 (0.42, 4.87) | 0.610 | | | 206/844 | 0.47 (0.05, 4.89) | 0.530 | | |
| ≤29.9 | 108/21556 | 1.76 (1.05, 2.95) | 0.029 | | | 595/3443 | 1.24 (0.08, 19.69) | 0.640 | | |
| >29.9 | 284/35862 | 2.27 (1.63, 3.16) | <0.001 | | | 850/6883 | 10.48 (1.20, 91.25) | 0.001 | | |
| Metabolic syndrome | | | | | 0.008 | | | | | 0.890 |
| 3 MetS component traits | 191/40293 | 3.07 (2.04, 4.61) | <0.001 | | | 1056/7439 | 2.64 (0.62, 11.26) | 0.120 | | |
| 4or5 MetS component traits | 224/20130 | 1.53 (1.07, 2.17) | 0.012 | | | 595/3731 | 3.62 (0.38, 34.13) | 0.091 | | |

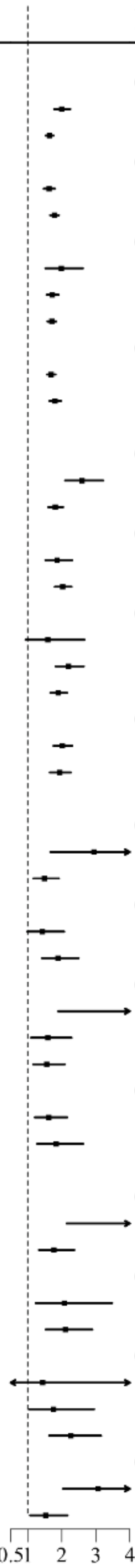


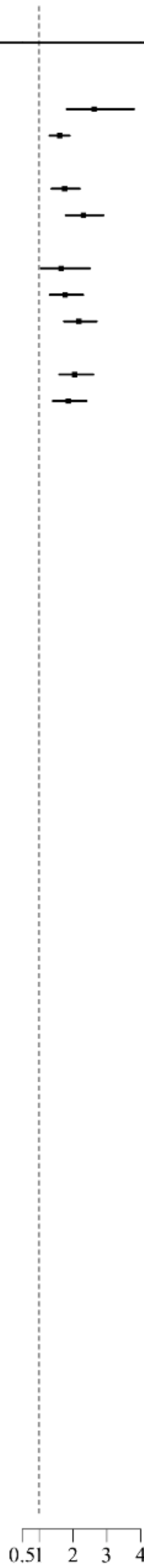


**Supplementary Figure 4. Stratified analyses of the association between social environments with all-cause and cause-specific mortality in UK Biobank and US NHANES.** The dots indicate the HRs comparing unfavorable versus favorable groups of social environments, and the horizontal lines indicate the 95%CIs. Model adjusted for age, sex, healthy lifestyle score, body mass index, the number of metabolic components traits, and prevalence of CVD, CBVD, CKD, diabetes, cancer and hypertension. The strata variable was not included in the model when stratified by itself. HR, hazard ratio; CI, confidence interval; CVD, cardiovascular disease; CBVD, cerebrovascular disease; CKD, chronic kidney disease; MetS, metabolic syndrome; NHANES, National Health and Nutrition Examination Survey.

| UK Biobank | | | | |
|---|---|---|---|---|
| Subgroup | Case/Total | HR(95%CI) | P for trend | P for interaction |
| **CVD incidence** | | | | |
| Age | | | | 0.001 |
| <60 | 4929/30696 | 1.42 (1.33, 1.52) | 0.007 | |
| ≥60 | 9376/29727 | 1.22 (1.16, 1.29) | 0.016 | |
| Sex | | | | 0.060 |
| Female | 4956/28492 | 1.37 (1.27, 1.47) | 0.003 | |
| Male | 9349/31931 | 1.25 (1.19, 1.33) | 0.021 | |
| Body mass index, kg/m2 | | | | 0.810 |
| <25 | 635/3005 | 1.23 (1.00, 1.51) | 0.150 | |
| ≤29.9 | 4820/21556 | 1.33 (1.23, 1.43) | 0.002 | |
| >29.9 | 8850/35862 | 1.28 (1.21, 1.35) | 0.074 | |
| Metabolic syndrome | | | | 0.820 |
| 3 MetS component traits | 8699/40293 | 1.30 (1.23, 1.37) | 0.001 | |
| 4or5 MetS component traits | 5606/20130 | 1.30 (1.21, 1.40) | 0.170 | |
| **CBVD incidence** | | | | |
| Age | | | | <0.001 |
| <60 | 1022/30696 | 1.79 (1.53, 2.09) | 0.200 | |
| ≥60 | 2196/29727 | 1.33 (1.21, 1.47) | 0.002 | |
| Sex | | | | 0.110 |
| Female | 1277/28492 | 1.48 (1.29, 1.69) | 0.012 | |
| Male | 1941/31931 | 1.45 (1.30, 1.61) | 0.017 | |
| Body mass index, kg/m2 | | | | 0.540 |
| <25 | 153/3005 | 1.47 (1.01, 2.14) | 0.011 | |
| ≤29.9 | 1142/21556 | 1.49 (1.30, 1.71) | 0.011 | |
| >29.9 | 1923/35862 | 1.43 (1.28, 1.59) | 0.120 | |
| Metabolic syndrome | | | | 0.800 |
| 3 MetS component traits | 1914/40293 | 1.47 (1.33, 1.64) | 0.036 | |
| 4or5 MetS component traits | 1304/20130 | 1.45 (1.27, 1.66) | 0.005 | |
| **CKD incidence** | | | | |
| Age | | | | <0.001 |
| <60 | 1633/30696 | 1.55 (1.35, 1.77) | 0.067 | |
| ≥60 | 4945/29727 | 1.28 (1.18, 1.38) | 0.065 | |
| Sex | | | | 0.660 |
| Female | 2991/28492 | 1.43 (1.29, 1.59) | 0.036 | |
| Male | 3587/31931 | 1.28 (1.17, 1.40) | 0.140 | |
| Body mass index, kg/m2 | | | | 0.960 |
| <25 | 289/3005 | 1.47 (1.04, 2.07) | 0.083 | |
| ≤29.9 | 2047/21556 | 1.35 (1.20, 1.52) | 0.086 | |
| >29.9 | 4242/35862 | 1.33 (1.22, 1.45) | 0.170 | |
| Metabolic syndrome | | | | 0.910 |
| 3 MetS component traits | 3709/40293 | 1.37 (1.25, 1.50) | 0.560 | |
| 4or5 MetS component traits | 2869/20130 | 1.33 (1.20, 1.48) | 0.003 | |

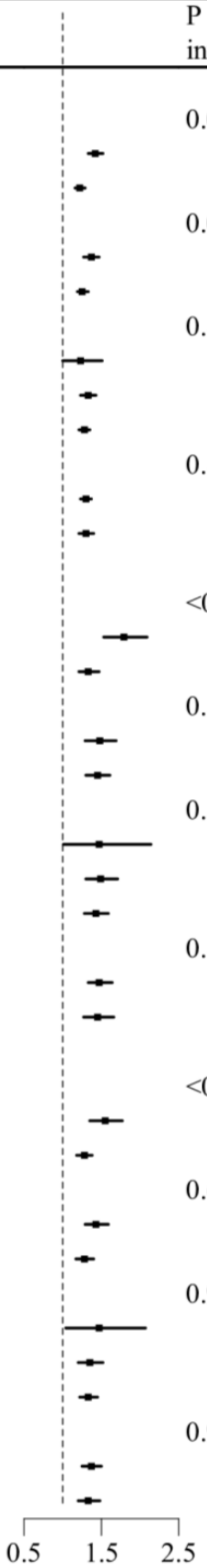


**Supplementary Figure 5. Stratified analyses of the association between social environments and diseases incidence in UK Biobank.** The dots indicate the HRs comparing unfavorable versus favorable groups of social environments, and the horizontal lines indicate the 95%CIs. Model adjusted for age, sex, healthy lifestyle score, body mass index, the number of metabolic components traits, and prevalence of CVD, CBVD, CKD, diabetes, cancer and hypertension. The strata variable and corresponding disease was not included in the model when stratified by itself. HR, hazard ratio; CI, confidence interval; CVD, cardiovascular disease; CBVD, cerebrovascular disease; CKD, chronic kidney disease; MetS, metabolic syndrome.

**Supplementary Table 1. Codes used to identify outcomes**

| Outcomes | UK Biobank | US NHANES |
|---|---|---|
| Mortality | | |
| All-cause mortality | -- | -- |
| CVD mortality | I00-I09; I11; I13; I20-I51 | 054-068 |
| CBVD mortality | I60-I69 | 070 |
| CKD mortality | N18, N18.1, N18.2, N18.3, N18.4, N18.5, N18.9 | 097-101 |
| Disease incidence | | |
| CVD incidence | I00-I09; I11; I13; I20-I51 | -- |
| CBVD incidence | I60-I69 | -- |
| CKD incidence | N18, N18.1, N18.2, N18.3, N18.4, N18.5, N18.9 | -- |

CVD, cardiovascular disease; CBVD, cerebrovascular disease; CKD, chronic kidney disease; NHANES, National Health and Nutrition Examination Survey.

**Supplementary Table 2. Assessment of social environments in UK Biobank and US NHANES**

| Domain | Social environments | UK Biobank | US NHANES |
|---|---|---|---|
| | | Definition (0= advantaged level, 1= disadvantaged level) | |
| Socioeconomic status | Family income level | 0: Average total household income before tax is more than £31,000;<br>1: Less than £31,000 | 0: family poverty / income ratio ≥300%;<br>1: family poverty / income ratio <300% |
| | Employment status | 0: Employed, retired or student;<br>1: Unemployed | 0: Employed, retired or student;<br>1: Unemployed |
| | Education attainment | 0: Highest education level is college or above;<br>1: Lower than college | 0: College graduate;<br>1: Less than college graduate |
| | Townsend deprivation index | 0: Below the median of Townsend deprivation index;<br>1: Above the median | ···- |
| | Food security | - | 0: Full food security (0 affirmative);<br>1: Marginal low, or very low (1-10 affirmative) |
| Social capital | Marital status | - | 0: Married or living with a partner;<br>1: Not married nor living with a partner |
| | Living alone/with partners | 0: Living with partners;<br>1: Living alone | - |
| | Social isolation | 0: Visit friend/family or have them visit you more than once a week;<br>1: Once a week or less often | - |
| | Social support | 0: Able to confide in anyone close to you at least once a week;<br>1: Once a week or less often | - |
| | Race/ethnicity | 0: Others;<br>1: Black race | 0: Others;<br>1: Black race |
| | Social activity | 0: Attend any group activities once a week or more often;<br>1: Less often than once a week | - |
| | Health insurance coverage | - | 0: Private insurance; |

| | | | |
|---|---|---|---|
| Healthcare access and quality | | | 1: Government or no insurance |
| | Health care quality | 0: Below the median of healthcare deprivation score;<br>1: Above the median | 0: Routine place to go for healthcare;<br>1: No place, or Emergency room/hospital/other |
| Neighborhood and built environment | Accommodation stability | 0: Own home;<br>1: Rent or other arrangement | 0: Own home;<br>1: Rent or other arrangement |
| | Natural environment | 0: Above the median of percentage of home location buffer classed as natural land;<br>1: Below the median | - |
| | Greenspace | 0: Above the median of percentage of home location buffer classed as greenspace;<br>1: Below the median | - |
| | Local crime rate | 0: Below the median of crime score;<br>1: Above the median | - |

-, the variable was not included in this cohort. NHANES, National Health and Nutrition Examination Survey.

**Supplementary Table 3. The weighted scores of social environments in UK Biobank and US NHANES**

| Domain | Social environments | UK Biobank | US NHANES |
|---|---|---|---|
| Socioeconomic status | Family income level | 0.389 | 0.505 |
| | Employment status | 0.626 | 0.587 |
| | Education attainment | 0.176 | 0.183 |
| | Townsend deprivation index | 0.275 | - |
| | Food security | - | 0.499 |
| Social capital | Marital status | - | 0.441 |
| | Living alone/with partners | 0.334 | - |
| | Social isolation | 0.048 | - |
| | Social support | 0.188 | - |
| | Race/ethnicity | 0.155 | 0.065 |
| | Social activity | 0.169 | - |
| Healthcare access and quality | Health insurance coverage | - | 0.421 |
| | Health care quality | 0.286 | 0.458 |
| Neighborhood and built environment | Accommodation stability | 0.185 | 0.258 |
| | Natural environment | 0.129 | - |
| | Greenspace | 0.093 | - |
| | Local crime rate | 0.174 | - |

-, the variable was not included in this cohort. NHANES, National Health and Nutrition Examination Survey.

**Supplementary Table 4. The weighted scores of healthy lifestyle in UK Biobank and US NHANES**

| Healthy lifestyle | UK Biobank | US NHANES |
|---|---|---|
| Cigarette smoking | 0.382 | 0.372 |
| Alcohol consumption | 0.076 | 0.106 |
| Physical activity | 0.066 | 0.162 |
| Diet | 0.036 | 0.174 |

NHANES, National Health and Nutrition Examination Survey.

**Supplementary Table 5. Codes used to identify prevalent comorbidities at baseline**

| Prevalent disease | UK Biobank | US NHANES |
|---|---|---|
| CVD | I00-I09; I11; I13; I20-I51 | MCQ160B, MCQ160C, MCQ160D, MCQ160E, MCQ160F |
| CBVD | I60-I69 | MCQ160f |
| CKD | N18, N18.1, N18.2, N18.3, N18.4, N18.5, N18.9 | LBXSCR, URXUMA, URXUCR |
| Diabetes | E10-E14 | LBXGLU, LBXGLT, DIQ010, DIQ050 |
| Cancer | C00-C99 | MCQ220 |
| Hypertension | 6150,6177,6153,4080,4079,93,94 | BPQ020, BPQ050A, BPXSAR, BPXDAR |

The prevalence of comorbidities was identified by ICD-10 codes, information from questionnaire and physical measure in UK Biobank and information from questionnaire in US NHANES. CVD, cardiovascular disease; CBVD, cerebrovascular disease; CKD, chronic kidney disease. ICD-10, The International Statistical Classification of Diseases and Related Health Problems 10th Revision; NHANES, National Health and Nutrition Examination Survey.

**Supplementary Table 6. Percentage of participants with disadvantaged level of each social environments***

| | UK Biobank cohort | | | | US NHANES cohort | | | |
|---|---|---|---|---|---|---|---|---|
| | Favorable | Medium | Unfavorable | Total population | Favorable | Medium | Unfavorable | Total population |
| Weighted social environments score range | ≤3.79 | 3.79-6.33 | >6.33 | | ≤1.79 | 1.79-4.08 | >4.08 | |
| Low household income | 4975(24.66) | 10787(53.63) | 17771(88.25) | 33533(55.50) | 837(20.81) | 2502(72.06) | 3558(96.79) | 6897(61.75) |
| Unemployed | 49(0.24) | 503(2.5) | 4467(22.18) | 5019(8.31) | 0(0) | 485(13.97) | 1848(50.27) | 2333(20.89) |
| Low education attainment | 11899(58.99) | 14837(73.76) | 17178(85.31) | 43914(72.68) | 1185(29.46) | 1436(41.36) | 2192(59.63) | 4813(43.09) |
| Low-income quality | 2571(12.75) | 10841(53.9) | 17830(88.54) | 31242(51.71) | - | - | - | - |
| Food insecurity | - | - | - | - | 88(2.19) | 766(22.06) | 2292(62.35) | 3146(28.16) |
| Living alone | 551(2.73) | 3131(15.57) | 8662(43.02) | 12344(20.43) | - | - | - | - |
| Social isolation | 11326(56.15) | 10990(54.64) | 11098(55.11) | 33414(55.30) | - | - | - | - |
| Unmarried | - | - | - | - | 515(12.8) | 1402(40.38) | 2345(63.79) | 4262(38.16) |
| Lack of social support | 4828(23.94) | 7331(36.45) | 10995(54.6) | 23154(38.32) | - | - | - | - |
| Black race | 28(0.14) | 94(0.47) | 621(3.08) | 743(1.23) | 630(15.66) | 700(20.16) | 922(25.08) | 2252(20.16) |
| Social inactive | 4966(24.62) | 6871(34.16) | 8626(42.84) | 20463(33.87) | - | - | - | - |
| Difficulty in access to healthcare | 2692(13.35) | 12264(60.97) | 18318(90.97) | 33274(55.07) | 89(2.21) | 388(11.18) | 995(27.07) | 1472(13.18) |
| Lack of health insurance | - | - | - | - | 389(9.67) | 1348(38.82) | 3134(85.26) | 4871(43.61) |
| Accommodation instability | 7796(38.65) | 9014(44.81) | 12431(61.73) | 29241(48.39) | 199(4.95) | 1140(32.83) | 2278(61.97) | 3617(32.38) |
| Lack of natural environment | 4915(24.37) | 10039(49.91) | 14400(71.51) | 29354(48.58) | - | - | - | - |
| Lack of Greenspace | 5278(26.17) | 10005(49.74) | 14133(70.18) | 29416(48.68) | - | - | - | - |
| High local crime rate | 3131(15.52) | 9829(48.86) | 15512(77.03) | 28472(47.12) | - | - | - | - |

*, Data are presented as n (%). -, the variable was not included in this cohort. NHANES, National Health and Nutrition Examination Survey.

**Supplementary Table 7. Baseline characteristics of participants included and excluded from analyses due to missing information**

| | UK Biobank cohort | | US NHANES cohort | |
|---|---|---|---|---|
| | Included (n=60423) | Excluded (n=36566) | Included (n=11170) | Excluded (n=11784) |
| Age, years, mean (SD) | 57.71(7.69) | 58.51(7.67) | 54.25(16.64) | 54.04(17.53) |
| Sex, n (%) | | | | |
| Women | 28492(47.15) | 20070(54.89) | 6387(57.18) | 6765(57.41) |
| Men | 31931(52.85) | 16496(45.11) | 4783(42.82) | 5019(42.59) |
| Race, n (%) | | | | |
| White | 57699(95.49) | 33091(91.84) | 5696(50.99) | 4887(41.47) |
| Black | 743(1.23) | 637(1.77) | 2252(20.16) | 2458(20.86) |
| Other | 1981(3.28) | 2303(6.39) | 3222(28.85) | 4439(37.67) |
| Body Mass Index, kg/m$^2$, mean (SD) | 31.48(4.77) | 31.55(4.88) | 32.68(6.69) | 31.84(6.34) |
| Smoking status, n (%) | | | | |
| Never | 29497(48.82) | 17839(69.73) | 5851(52.38) | 6126(52.07) |
| Past | 23987(39.70) | 3179(12.43) | 3251(29.10) | 3157(26.84) |
| Current | 6939(11.48) | 4566(17.85) | 2068(18.51) | 2481(21.09) |
| Drinking, n (%) | | | | |
| ≤2 drinks/week | 22298(36.9) | 10543(29.05) | 6653(59.56) | 6328(61.62) |
| >2 drinks/week | 38125(63.1) | 25750(70.95) | 4517(40.44) | 3942(38.38) |
| Physical activity, n (%) | | | | |
| Inactive | 33612(55.63) | 16532(57.18) | 6824(61.09) | 6083(72.22) |
| Insufficiently active | 9919(16.42) | 4448(15.38) | 2474(22.15) | 1306(15.51) |
| Active | 16892(27.96) | 7933(27.44) | 1872(16.76) | 1034(12.28) |
| Diet, n (%) | | | | |
| Unhealthy | 35980(59.55) | 18378(52.91) | 6702(60.00) | 5754(65.21) |
| Healthy | 24443(40.45) | 16355(47.09) | 4468(40.00) | 3070(34.79) |

NHANES, National Health and Nutrition Examination Survey.

**Supplementary Table 8. Weighted and unweighted PAF for social environments domains**

| Domains | UK Biobank | | | US NHANES | | |
|---|---|---|---|---|---|---|
| | Unweighted PAF | Communality | Weighted PAF | Unweighted PAF | Communality | Weighted PAF |
| All-cause mortality | | | | | | |
| Health care | 0.044 | 0.355 | 0.032 | 0.113 | 0.296 | 0.080 |
| Neighborhood and Built Environment | 0.016 | 0.274 | 0.011 | - | - | - |
| Social capital | 0.076 | 0.069 | 0.055 | 0.138 | 0.124 | 0.098 |
| Socioeconomic status | 0.099 | 0.302 | 0.072 | 0.095 | 0.303 | 0.067 |
| Overall weighted PAF | | | 0.170 | | | 0.241 |
| CVD mortality | | | | | | |
| Health care | - | - | - | 0.098 | 0.296 | 0.076 |
| Neighborhood and Built Environment | 0.023 | 0.274 | 0.016 | - | - | - |
| Social capital | 0.265 | 0.069 | 0.183 | 0.124 | 0.124 | 0.097 |
| Socioeconomic status | 0.352 | 0.302 | 0.243 | - | - | - |
| Overall weighted PAF | | | 0.442 | | | 0.161 |
| CBVD mortality | | | | | | |
| Health care | 0.015 | 0.355 | 0.011 | -0.032 | 0.296 | -0.025 |
| Neighborhood and Built Environment | - | - | - | -0.044 | 0.277 | -0.034 |
| Social capital | 0.223 | 0.069 | 0.162 | 0.009 | 0.124 | 0.007 |
| Socioeconomic status | 0.269 | 0.302 | 0.195 | 0.122 | 0.303 | 0.094 |
| Overall weighted PAF | | | 0.353 | | | 0.042 |
| CKD mortality | | | | | | |
| Health care | 0.139 | 0.355 | 0.089 | 0.250 | 0.296 | 0.172 |
| Neighborhood and Built Environment | 0.113 | 0.274 | 0.073 | - | 0.280 | - |

| | | | | | | |
|---|---|---|---|---|---|---|
| Social capital | 0.224 | 0.069 | 0.144 | 0.300 | 0.124 | 0.207 |
| Socioeconomic status | 0.176 | 0.302 | 0.114 | 0.255 | 0.303 | 0.175 |
| Overall weighted PAF | | | 0.420 | | | 0.441 |

-, indicated that the PAF value is too small to hold practical statistical significance. Model adjusted for age, sex, healthy lifestyle score, body mass index, the number of metabolic components traits, and prevalence of CVD, CBVD, CKD, diabetes, cancer and hypertension. Weighted PAF was calculated after considering the overlap between risk factors. PAF, population attributable fraction; CVD, cardiovascular disease; CBVD, cerebrovascular disease; CKD, chronic kidney disease; NHANES, National Health and Nutrition Examination Survey.

**Supplementary Table 9. Associations of the social environments with all-cause and cause-specific mortality among adults with MetS in UK Biobank cohort: sensitivity analyses**

| | All-cause mortality | CVD mortality | CBVD mortality | CKD mortality |
|---|---|---|---|---|
| | HR (95%CI) | HR (95%CI) | HR (95%CI) | HR (95%CI) |
| **Sensitivity analyses 1: excluding events that occurred within the first two years of follow-up** | | | | |
| Favorable social environments | 1.00 (ref.) | 1.00 (ref.) | 1.00 (ref.) | 1.00 (ref.) |
| Medium social environments | 1.15 (1.07, 1.22) | 1.23 (1.10, 1.39) | 1.18 (0.93, 1.51) | 1.42 (1.07, 1.90) |
| Unfavorable social environments | 1.72 (1.61, 1.83) | 1.94 (1.74, 2.17) | 1.62 (1.29, 2.05) | 2.06 (1.57, 2.70) |
| **Sensitivity analyses 2: excluding individuals with prevalent CVD, CBVD, CKD, diabetes or cancer** | | | | |
| Favorable social environments | 1.00 (ref.) | 1.00 (ref.) | 1.00 (ref.) | 1.00 (ref.) |
| Medium social environments | 1.11 (1.02, 1.22) | 1.17 (0.98, 1.40) | 1.45 (1.00, 2.10) | 1.80 (0.96, 3.37) |
| Unfavorable social environments | 1.66 (1.52, 1.80) | 1.86 (1.58, 2.20) | 1.68 (1.16, 2.45) | 2.76 (1.53, 5.01) |
| **Sensitivity analyses 3: multiple imputation** | | | | |
| Favorable social environments | 1.00 (ref.) | 1.00 (ref.) | 1.00 (ref.) | 1.00 (ref.) |
| Medium social environments | 1.22 (1.14, 1.30) | 1.27 (1.15, 1.40) | 1.36 (1.07, 1.73) | 1.55 (1.16, 2.08) |
| Unfavorable social environments | 1.89 (1.78, 2.01) | 2.09 (1.91, 2.30) | 1.85 (1.47, 2.32) | 2.38 (1.80, 3.14) |
| **Sensitivity analyses 4: using unweighted social environment scores** | | | | |
| Favorable social environments | 1.00 (ref.) | 1.00 (ref.) | 1.00 (ref.) | 1.00 (ref.) |
| Medium social environments | 1.18 (1.11, 1.25) | 1.14 (1.03, 1.27) | 1.10 (0.88, 1.38) | 1.23 (0.95, 1.60) |
| Unfavorable social environments | 1.49 (1.41, 1.58) | 1.63 (1.49, 1.80) | 1.37 (1.12, 1.69) | 1.74 (1.38, 2.20) |

Model adjusted for age, sex, healthy lifestyle score, body mass index, the number of metabolic components traits, and prevalence of CVD, CBVD, CKD, diabetes, cancer and hypertension. CVD, cardiovascular disease; CBVD, cerebrovascular disease; CKD, chronic kidney disease; HR, hazard ratio; CI, confidence interval; MetS, metabolic syndrome.

**Supplementary Table 10. Associations of the social environments with mortality among adults with MetS in US NHANES cohort: sensitivity analyses**

| | All-cause mortality | CVD mortality | CBVD mortality | CKD mortality |
|---|---|---|---|---|
| | HR (95%CI) | HR (95%CI) | HR (95%CI) | HR (95%CI) |
| **Sensitivity analyses 1: excluding events that occurred within the first two years of follow-up** | | | | |
| Favorable social environments | 1.00 (ref.) | 1.00 (ref.) | 1.00 (ref.) | 1.00 (ref.) |
| Medium social environments | 1.47 (1.28,1.68) | 1.12 (0.88, 1.42) | 1.56 (0.86, 2.81) | 4.72 (1.58, 14.16) |
| Unfavorable social environments | 2.25 (1.87,2.69) | 1.34 (1.01, 1.77) | 1.06 (0.47, 2.38) | 2.50 (0.69, 9.11) |
| **Sensitivity analyses 2: excluding individuals with prevalent CVD, CBVD, CKD, diabetes or cancer** | | | | |
| Favorable social environments | 1.00 (ref.) | 1.00 (ref.) | 1.00 (ref.) | - |
| Medium social environments | 1.65 (1.23,2.21) | 1.36 (0.85, 2.18) | 1.24 (0.38, 4.01) | - |
| Unfavorable social environments | 3.16 (2.23,4.46) | 1.87 (1.12, 3.12) | 1.43 (0.33, 6.17) | - |
| **Sensitivity analyses 3: multiple imputation** | | | | |
| Favorable social environments | 1.00 (ref.) | 1.00 (ref.) | 1.00 (ref.) | 1.00 (ref.) |
| Medium social environments | 1.46 (1.35,1.58) | 1.20 (1.04, 1.39) | 1.07 (0.79, 1.45) | 1.64 (0.99, 2.72) |
| Unfavorable social environments | 2.02 (1.80,2.25) | 1.32 (1.12, 1.54) | 1.27 (0.90, 1.77) | 1.64 (0.97, 2.76) |
| **Sensitivity analyses 4: using unweighted social environment scores** | | | | |
| Favorable social environments | 1.00 (ref.) | 1.00 (ref.) | 1.00 (ref.) | 1.00 (ref.) |
| Medium social environments | 1.35 (1.18, 1.54) | 1.07 (1.06, 1.08) | 1.08 (1.05, 1.11) | 1.04 (1.01, 1.08) |
| Unfavorable social environments | 1.86 (1.53, 2.25) | 1.42 (1.17, 1.71) | 1.03 (0.65, 1.64) | 1.68 (0.87, 3.24) |
| **Sensitivity analyses 5: among individuals aged 40 years or older** | | | | |
| Favorable social environments | 1.00 (ref.) | 1.00 (ref.) | 1.00 (ref.) | 1.00 (ref.) |
| Medium social environments | 1.43 (1.27, 1.62) | 1.17 (0.93, 1.46) | 1.59 (0.93, 2.72) | 5.75 (1.94, 17.03) |
| Unfavorable social environments | 2.31 (1.95, 2.73) | 1.55 (1.20, 2.01) | 1.13 (0.55, 2.33) | 3.37 (1.03, 11.01) |

-, Due to the small sample size, this value is not of reference significance. Model adjusted for age, sex, healthy lifestyle score, body mass index, the number of metabolic components traits, and prevalence of CVD, CBVD, CKD, diabetes, cancer and hypertension. CVD, cardiovascular disease; CBVD, cerebrovascular disease; CKD, chronic kidney disease; HR, hazard ratio; CI, confidence interval; MetS, metabolic syndrome; NHANES, National Health and Nutrition Examination Survey.

**Supplementary Table 11. Estimates of year of life loss based on combined social environments among adults with MetS in the UK Biobank and US NHANES**

| | UK Biobank | | | US NHANES | | |
|---|---|---|---|---|---|---|
| | Favorable | Medium | Unfavorable | Favorable | Medium | Unfavorable |
| Weighted social environments score range | ≤3.79 | 3.79-6.33 | >6.33 | ≤1.79 | 1.79-4.08 | >4.08 |
| At age 45 years | Ref. | 0.65 (0.57, 0.74) | 3.49 (3.40, 3.57) | Ref. | 3.79 (3.53, 4.05) | 7.81 (7.56, 8.07) |
| At age 55 years | Ref. | 0.63 (0.55, 0.72) | 3.39 (3.30, 3.47) | Ref. | 3.56 (3.32, 3.80) | 7.26 (7.03, 7.50) |
| At age 65 years | Ref. | 0.58 (0.50, 0.65) | 3.08 (3.00, 3.16) | Ref. | 3.09 (2.88, 3.30) | 6.20 (6.00, 6.40) |
| At age 75 years | Ref. | 0.45 (0.38, 0.51) | 2.37 (2.31, 2.43) | Ref. | 2.34 (2.18, 2.51) | 4.57 (4.42, 4.72) |

MetS, metabolic syndrome; NHANES, National Health and Nutrition Examination Survey.

**Supplementary Table 12. Estimates of life expectancy based on combined social environments among adults with MetS in the UK Biobank and US NHANES**

| | UK Biobank | | | US NHANES | | |
|---|---|---|---|---|---|---|
| | Favorable | Medium | Unfavorable | Favorable | Medium | Unfavorable |
| Weighted social environments score range | ≤3.79 | 3.79-6.33 | >6.33 | ≤1.79 | 1.79-4.08 | >4.08 |
| At age 45 years | 37.92 (37.86, 37.98) | 37.27 (37.21, 37.33) | 34.43 (34.37, 34.50) | 36.82 (36.66, 36.99) | 33.03 (32.83, 33.23) | 29.01 (28.82, 29.20) |
| At age 55 years | 28.07 (28.01, 28.12) | 27.43 (27.37, 27.49) | 24.68 (24.62, 24.74) | 27.33 (27.17, 27.49) | 23.77 (23.58, 23.95) | 20.07 (19.89, 20.24) |
| At age 65 years | 18.61 (18.56, 18.67) | 18.04 (17.98, 18.09) | 15.54 (15.48, 15.59) | 18.53 (18.39, 18.67) | 15.44 (15.28, 15.60) | 12.33 (12.19, 12.47) |
| At age 75 years | 10.30 (10.26, 10.34) | 9.86 (9.81, 9.90) | 7.93 (7.89, 7.98) | 11.06 (10.94, 11.17) | 8.71 (8.59, 8.83) | 6.49 (6.39, 6.58) |

MetS, metabolic syndrome; NHANES, National Health and Nutrition Examination Survey.

**Supplementary Table 13. Associations of the social environments with diseases incidence among adults with MetS in UK Biobank cohort: sensitivity analyses**

| | CVD incidence | CBVD incidence | CKD incidence |
|---|---|---|---|
| | HR (95%CI) | HR (95%CI) | HR (95%CI) |
| **Sensitivity analyses 1: excluding events that occurred within the first two years of follow-up** | | | |
| Favorable social environments | 1.00 (ref.) | 1.00 (ref.) | 1.00 (ref.) |
| Medium social environments | 1.08 (1.03, 1.13) | 1.13 (1.03, 1.23) | 1.06 (0.99, 1.14) |
| Unfavorable social environments | 1.28 (1.23, 1.34) | 1.44 (1.32, 1.57) | 1.30 (1.21, 1.40) |
| **Sensitivity analyses 2: excluding individuals with prevalent CVD, CBVD, CKD, diabetes or cancer** | | | |
| Favorable social environments | 1.00 (ref.) | 1.00 (ref.) | 1.00 (ref.) |
| Medium social environments | 1.06 (1.01, 1.12) | 1.11 (0.99, 1.24) | 1.08 (0.98, 1.20) |
| Unfavorable social environments | 1.25 (1.19, 1.31) | 1.51 (1.35, 1.69) | 1.36 (1.23, 1.50) |
| **Sensitivity analyses 3: multiple imputation** | | | |
| Favorable social environments | 1.00 (ref.) | 1.00 (ref.) | 1.00 (ref.) |
| Medium social environments | 1.11 (1.06, 1.16) | 1.26 (1.16, 1.36) | 1.09 (1.03, 1.16) |
| Unfavorable social environments | 1.38 (1.32, 1.44) | 1.59 (1.47, 1.72) | 1.36 (1.28, 1.44) |
| **Sensitivity analyses 4: using unweighted social environment scores** | | | |
| Favorable social environments | 1.00 (ref.) | 1.00 (ref.) | 1.00 (ref.) |
| Medium social environments | 1.06 (1.01, 1.10) | 1.10 (1.01, 1.20) | 1.07 (1.00, 1.15) |
| Unfavorable social environments | 1.21 (1.16, 1.26) | 1.31 (1.21, 1.41) | 1.23 (1.15, 1.31) |

Model adjusted for age, sex, healthy lifestyle score, body mass index, the number of metabolic components traits, and prevalence of CVD, CBVD, CKD, diabetes, cancer and hypertension. Prevalence of corresponding disease was not included for the analysis of diseases incidence. CVD, cardiovascular disease; CBVD, cerebrovascular disease; CKD, chronic kidney disease; HR, hazard ratio; CI, confidence interval; MetS, metabolic syndrome.

**Supplementary Table 14. The association of lifestyle with all-cause and cause-specific mortality by social environments among adults with MetS in UK Biobank cohort**

| | All-cause mortality | CVD mortality | CBVD mortality | CKD mortality |
|---|---|---|---|---|
| | HR (95%CI) | HR (95%CI) | HR (95%CI) | HR (95%CI) |
| Favorable social environments | | | | |
| Unhealthy lifestyle | 1.00 (ref.) | 1.00 (ref.) | 1.00 (ref.) | 1.00 (ref.) |
| Medium healthy lifestyle | 0.85 (0.77, 0.95) | 0.90 (0.74, 1.09) | 1.19 (0.76, 1.86) | 0.71 (0.43, 1.20) |
| Healthy lifestyle | 0.72 (0.64, 0.82) | 0.65 (0.51, 0.82) | 1.18 (0.71, 1.94) | 0.46 (0.23, 0.92) |
| $P_{trend}$ | <0.001 | <0.001 | 0.500 | 0.023 |
| Medium social environments | | | | |
| Unhealthy lifestyle | 1.00 (ref.) | 1.00 (ref.) | 1.00 (ref.) | 1.00 (ref.) |
| Medium healthy lifestyle | 0.73 (0.66, 0.80) | 0.76 (0.65, 0.90) | 0.87 (0.62, 1.22) | 0.80 (0.54, 1.17) |
| Healthy lifestyle | 0.61 (0.54, 0.69) | 0.58 (0.46, 0.72) | 0.57 (0.35, 0.91) | 0.55 (0.32, 0.95) |
| $P_{trend}$ | <0.001 | <0.001 | 0.019 | 0.025 |
| Unfavorable social environments | | | | |
| Unhealthy lifestyle | 1.00 (ref.) | 1.00 (ref.) | 1.00 (ref.) | 1.00 (ref.) |
| Medium healthy lifestyle | 0.72 (0.67, 0.78) | 0.70 (0.62, 0.80) | 0.93 (0.70, 1.22) | 0.71 (0.53, 0.96) |
| Healthy lifestyle | 0.62 (0.55, 0.69) | 0.60 (0.49, 0.72) | 0.54 (0.34, 0.86) | 0.63 (0.40, 0.99) |
| $P_{trend}$ | <0.001 | <0.001 | 0.014 | 0.013 |
| $P_{interaction}$ | 0.047 | 0.630 | 0.016 | 0.300 |
| RERI | -0.43 (-0.62, -0.23) | -0.44 (-0.79, -0.09) | -1.23 (-2.29, -0.16) | 0.00 (-0.72, 0.72) |

Model adjusted for age, sex, healthy lifestyle score, body mass index, the number of metabolic components traits, and prevalence of CVD, CBVD, CKD, diabetes, cancer and hypertension. HR, hazard ratio; CI, confidence interval; MetS, metabolic syndrome; CVD, cardiovascular disease; CBVD, cerebrovascular disease; CKD, chronic kidney disease; RERI: relative excess risk due to interaction.

**Supplementary Table 15. The association of lifestyle and diseases incidence by social environments among adults with MetS in UK Biobank cohort**

| | CVD incidence | CBVD incidence | CKD incidence |
|---|---|---|---|
| | HR (95%CI) | HR (95%CI) | HR (95%CI) |
| Favorable social environments | | | |
| Unhealthy lifestyle | 1.00 (ref.) | 1.00 (ref.) | 1.00 (ref.) |
| Medium healthy lifestyle | 0.87 (0.81, 0.94) | 1.07 (0.92, 1.24) | 0.92 (0.82, 1.04) |
| Healthy lifestyle | 0.87 (0.80, 0.94) | 0.96 (0.81, 1.14) | 0.78 (0.68, 0.90) |
| $P_{trend}$ | <0.001 | 0.650 | <0.001 |
| Medium social environments | | | |
| Unhealthy lifestyle | 1.00 (ref.) | 1.00 (ref.) | 1.00 (ref.) |
| Medium healthy lifestyle | 0.92 (0.86, 0.99) | 0.91 (0.79, 1.04) | 0.87 (0.78, 0.97) |
| Healthy lifestyle | 0.80 (0.74, 0.87) | 0.91 (0.78, 1.06) | 0.78 (0.68, 0.89) |
| $P_{trend}$ | <0.001 | 0.190 | <0.001 |
| Unfavorable social environments | | | |
| Unhealthy lifestyle | 1.00 (ref.) | 1.00 (ref.) | 1.00 (ref.) |
| Medium healthy lifestyle | 0.87 (0.82, 0.93) | 0.81 (0.72, 0.91) | 0.84 (0.77, 0.93) |
| Healthy lifestyle | 0.77 (0.71, 0.84) | 0.68 (0.58, 0.80) | 0.79 (0.69, 0.90) |
| $P_{trend}$ | <0.001 | <0.001 | <0.001 |
| $P_{interaction}$ | 0.053 | 0.006 | 0.640 |
| RERI | -0.16 (-0.28, -0.03) | -0.48 (-0.77, -0.19) | -0.02 (-0.21, 0.17) |

Model adjusted for age, sex, healthy lifestyle score, body mass index, the number of metabolic components traits, and prevalence of CVD, CBVD, CKD, diabetes, cancer and hypertension. Prevalence of corresponding disease was not included for the analysis of diseases incidence. CVD, cardiovascular disease; CBVD, cerebrovascular disease; CKD, chronic kidney disease; HR, hazard ratio; CI, confidence interval; MetS, metabolic syndrome; RERI, relative excess risk due to interaction.

**Supplementary Table 16. The association of lifestyle with all-cause and cause-specific mortality by social environments among adults with MetS in US NHANES cohort**

| | All-cause mortality | CVD mortality | CBVD mortality | CKD mortality |
|---|---|---|---|---|
| | HR (95%CI) | HR (95%CI) | HR (95%CI) | HR (95%CI) |
| Favorable social environments | | | | |
| Unhealthy lifestyle | 1.00 (ref.) | 1.00 (ref.) | 1.00 (ref.) | - |
| Medium healthy lifestyle | 0.77 (0.60, 0.98) | 1.20 (0.83, 1.73) | 0.75 (0.32, 1.74) | - |
| Healthy lifestyle | 0.73 (0.55, 0.95) | 0.96 (0.62, 1.50) | 0.62 (0.22, 1.73) | - |
| $P_{trend}$ | 0.016 | 1.000 | 0.340 | - |
| Medium social environments | | | | |
| Unhealthy lifestyle | 1.00 (ref.) | 1.00 (ref.) | 1.00 (ref.) | 1.00 (ref.) |
| Medium healthy lifestyle | 0.81 (0.65, 1.00) | 1.30 (0.93, 1.82) | 1.41 (0.65, 3.06) | 2.81 (0.98, 8.07) |
| Healthy lifestyle | 0.58 (0.43, 0.78) | 0.54 (0.32, 0.93) | 1.32 (0.48, 3.65) | 0.86 (0.17, 4.41) |
| $P_{trend}$ | <0.001 | 0.120 | 0.510 | 0.540 |
| Unfavorable social environments | | | | |
| Unhealthy lifestyle | 1.00 (ref.) | 1.00 (ref.) | 1.00 (ref.) | 1.00 (ref.) |
| Medium healthy lifestyle | 0.65 (0.51, 0.82) | 0.83 (0.56, 1.25) | 0.96 (0.35, 2.63) | 1.29 (0.41, 4.02) |
| Healthy lifestyle | 0.54 (0.39, 0.75) | 0.60 (0.34, 1.06) | 0.67 (0.16, 2.89) | 0.63 (0.08, 5.10) |
| $P_{trend}$ | <0.001 | 0.074 | 0.600 | 0.860 |
| $P_{interaction}$ | 0.206 | 0.250 | 0.680 | <0.001 |
| RERI | -0.89(-1.54, -0.23) | -0.71 (-1.69, 0.28) | 0.28 (-1.20, 1.77) | 0.50 (-1.27, 2.28) |

-, Due to the small sample size, this value is not of reference significance. Model adjusted for age, sex, healthy lifestyle score, body mass index, the number of metabolic components traits, and prevalence of CVD, CBVD, CKD, diabetes, cancer and hypertension. HR, hazard ratio; CI, confidence interval; MetS, metabolic syndrome; CVD, cardiovascular disease; CBVD, cerebrovascular disease; CKD, chronic kidney disease; NHANES, National Health and Nutrition Examination Survey; RERI, relative excess risk due to interaction.

**Supplementary Table 17. The association of lifestyle with all-cause and cause-specific mortality by social environments among adults with MetS in UK Biobank cohort: using unweighted healthy lifestyle score**

| | All-cause mortality | CVD mortality | CBVD mortality | CKD mortality |
|---|---|---|---|---|
| | HR (95%CI) | HR (95%CI) | HR (95%CI) | HR (95%CI) |
| Favorable social environments | | | | |
| 0 or 1 healthy behaviors | 1.00 (ref.) | 1.00 (ref.) | 1.00 (ref.) | 1.00 (ref.) |
| 2 healthy behaviors | 0.92 (0.83, 1.02) | 0.92 (0.76, 1.11) | 1.33 (0.88, 2.00) | 1.08 (0.66, 1.79) |
| 3 or 4 healthy behaviors | 0.86 (0.76, 0.97) | 0.76 (0.60, 0.97) | 1.08 (0.65, 1.80) | 0.88 (0.47, 1.66) |
| $P_{trend}$ | 0.013 | 0.029 | 0.600 | 0.770 |
| Medium social environments | | | | |
| 0 or 1 healthy behaviors | 1.00 (ref.) | 1.00 (ref.) | 1.00 (ref.) | 1.00 (ref.) |
| 2 healthy behaviors | 0.88 (0.79, 0.97) | 0.94 (0.80, 1.12) | 0.89 (0.62, 1.27) | 0.66 (0.43, 1.01) |
| 3 or 4 healthy behaviors | 0.75 (0.66, 0.84) | 0.79 (0.64, 0.96) | 0.76 (0.50, 1.16) | 0.84 (0.54, 1.32) |
| $P_{trend}$ | <0.001 | 0.024 | 0.200 | 0.310 |
| Unfavorable social environments | | | | |
| 0 or 1 healthy behaviors | 1.00 (ref.) | 1.00 (ref.) | 1.00 (ref.) | 1.00 (ref.) |
| 2 healthy behaviors | 0.83 (0.77, 0.90) | 0.82 (0.72, 0.94) | 1.10 (0.82, 1.49) | 1.15 (0.85, 1.56) |
| 3 or 4 healthy behaviors | 0.75 (0.68, 0.83) | 0.74 (0.63, 0.87) | 1.03 (0.73, 1.47) | 0.83 (0.57, 1.22) |
| $P_{trend}$ | <0.001 | <0.001 | 0.780 | 0.510 |
| $P_{interaction}$ | 0.046 | 0.770 | 0.690 | 0.900 |
| RERI | -0.34 (-0.53, -0.15) | -0.30 (-0.64, 0.04) | -0.15 (-0.98, 0.67) | -0.17 (-1.09, 0.75) |

Model adjusted for age, sex, healthy lifestyle score, body mass index, the number of metabolic components traits, and prevalence of CVD, CBVD, CKD, diabetes, cancer and hypertension. CVD, cardiovascular disease; CBVD, cerebrovascular disease; CKD, chronic kidney disease; HR, hazard ratio; CI, confidence interval; MetS, metabolic syndrome; RERI, relative excess risk due to interaction.

**Supplementary Table 18. The association of lifestyle and diseases incidence by social environments among adults with MetS in UK Biobank cohort: using unweighted healthy lifestyle score**

| | CVD incidence | CBVD incidence | CKD incidence |
|---|---|---|---|
| | HR (95%CI) | HR (95%CI) | HR (95%CI) |
| Favorable social environments | | | |
| 0 or 1 Healthy behaviors | 1.00 (ref.) | 1.00 (ref.) | 1.00 (ref.) |
| 2 Healthy behaviors | 0.94 (0.87, 1.01) | 0.96 (0.83, 1.11) | 1.12 (0.99, 1.26) |
| 3 or 4 Healthy behaviors | 0.96 (0.88, 1.04) | 0.90 (0.76, 1.06) | 1.02 (0.89, 1.17) |
| $P_{trend}$ | 0.200 | 0.210 | 0.540 |
| Medium social environments | | | |
| 0 or 1 Healthy behaviors | 1.00 (ref.) | 1.00 (ref.) | 1.00 (ref.) |
| 2 Healthy behaviors | 0.96 (0.90, 1.04) | 1.05 (0.92, 1.20) | 1.09 (0.97, 1.21) |
| 3 or 4 Healthy behaviors | 0.90 (0.83, 0.98) | 0.98 (0.84, 1.14) | 0.90 (0.80, 1.03) |
| $P_{trend}$ | 0.014 | 0.860 | 0.210 |
| Unfavorable social environments | | | |
| 0 or 1 Healthy behaviors | 1.00 (ref.) | 1.00 (ref.) | 1.00 (ref.) |
| 2 Healthy behaviors | 0.89 (0.83, 0.95) | 1.00 (0.89, 1.12) | 1.02 (0.93, 1.13) |
| 3 or 4 Healthy behaviors | 0.90 (0.83, 0.97) | 0.82 (0.71, 0.94) | 0.95 (0.84, 1.06) |
| $P_{trend}$ | 0.002 | 0.010 | 0.400 |
| $P_{interaction}$ | 0.360 | 0.440 | 0.600 |
| RERI | -0.08 (-0.20, 0.04) | -0.16 (-0.40, 0.08) | -0.07 (-0.27, 0.13) |

Model adjusted for age, sex, healthy lifestyle score, body mass index, the number of metabolic components traits, and prevalence of CVD, CBVD, CKD, diabetes, cancer and hypertension. Prevalence of corresponding disease was not included for the analysis of diseases incidence. CVD, cardiovascular disease; CBVD, cerebrovascular disease; CKD, chronic kidney disease; HR, hazard ratio; CI, confidence interval; MetS, metabolic syndrome; RERI, relative excess risk due to interaction.

**Supplementary Table 19. The association of lifestyle with all-cause and cause-specific mortality by social environments among adults with MetS in US NHANES cohort: using unweighted healthy lifestyle score**

| | All-cause mortality | CVD mortality | CBVD mortality | CKD mortality |
|---|---|---|---|---|
| | HR (95%CI) | HR (95%CI) | HR (95%CI) | HR (95%CI) |
| Favorable social environments | | | | |
| 0 or 1 Healthy behaviors | 1.00 (ref.) | 1.00 (ref.) | 1.00 (ref.) | - |
| 2 Healthy behaviors | 0.73 (0.57, 0.94) | 0.84 (0.59, 1.19) | 0.67 (0.29, 1.58) | - |
| 3 or 4 Healthy behaviors | 0.90 (0.68, 1.19) | 0.97 (0.63, 1.52) | 0.57 (0.19, 1.77) | - |
| $P_{trend}$ | 0.164 | 0.690 | 0.270 | - |
| Medium social environments | | | | |
| 0 or 1 Healthy behaviors | 1.00 (ref.) | 1.00 (ref.) | 1.00 (ref.) | 1.00 (ref.) |
| 2 Healthy behaviors | 0.76 (0.61, 0.95) | 1.20 (0.87, 1.66) | 0.79 (0.35, 1.77) | 2.63 (0.98, 7.08) |
| 3 or 4 Healthy behaviors | 0.68 (0.50, 0.93) | 0.63 (0.36, 1.10) | 1.31 (0.52, 3.27) | 0.50 (0.06, 3.88) |
| $P_{trend}$ | 0.003 | 0.330 | 0.770 | 0.620 |
| Unfavorable social environments | | | | |
| 0 or 1 Healthy behaviors | 1.00 (ref.) | 1.00 (ref.) | 1.00 (ref.) | 1.00 (ref.) |
| 2 Healthy behaviors | 0.61 (0.48, 0.78) | 0.73 (0.49, 1.09) | 1.05 (0.39, 2.81) | 0.36 (0.08, 1.60) |
| 3 or 4 Healthy behaviors | 0.63 (0.46, 0.86) | 0.68 (0.39, 1.18) | 0.76 (0.17, 3.46) | 0.42 (0.05, 3.27) |
| $P_{trend}$ | <0.001 | 0.085 | 0.760 | 0.230 |
| $P_{interaction}$ | 0.078 | 0.280 | 0.570 | <0.001 |
| RERI | -0.96 (-1.68, -0.24) | -0.60 (-1.54, 0.35) | 0.42 (-1.08, 1.92) | -0.68 (-4.48, 3.13) |

-, Due to the small sample size, this value is not of reference significance. Model adjusted for age, sex, healthy lifestyle score, body mass index, the number of metabolic components traits, and prevalence of CVD, CBVD, CKD, diabetes, cancer and hypertension. CVD, cardiovascular disease; CBVD, cerebrovascular disease; CKD, chronic kidney disease; HR, hazard ratio; CI, confidence interval; MetS, metabolic syndrome; NHANES, National Health and Nutrition Examination Survey; RERI, relative excess risk due to interaction.

**Supplementary Table 20. The association of lifestyle with all-cause and cause-specific mortality by social environments among adults aged≥ 40 years with MetS in US NHANES cohort**

| | All-cause mortality | CVD mortality | CBVD mortality | CKD mortality |
|---|---|---|---|---|
| | HR (95%CI) | HR (95%CI) | HR (95%CI) | HR (95%CI) |
| Favorable social environments | | | | |
| Unhealthy lifestyle | 1.00 (ref.) | 1.00 (ref.) | 1.00 (ref.) | - |
| Medium healthy lifestyle | 0.80 (0.63,1.03) | 1.25 (0.87, 1.81) | 0.75 (0.32, 1.74) | - |
| Healthy lifestyle | 0.74 (0.57, 0.97) | 1.00 (0.64, 1.56) | 0.62 (0.22, 1.73) | - |
| $P_{trend}$ | 0.023 | 0.870 | 0.340 | - |
| Medium social environments | | | | |
| Unhealthy lifestyle | 1.00 (ref.) | 1.00 (ref.) | 1.00 (ref.) | 1.00 (ref.) |
| Medium healthy lifestyle | 0.86 (0.69, 1.07) | 1.34 (0.95, 1.88) | 1.37 (0.62, 3.06) | 2.81 (0.98, 8.06) |
| Healthy lifestyle | 0.59 (0.44, 0.79) | 0.53 (0.31, 0.92) | 1.35 (0.49, 3.77) | 0.86 (0.17, 4.41) |
| $P_{trend}$ | <0.001 | 0.120 | 0.490 | 0.530 |
| Unfavorable social environments | | | | |
| Unhealthy lifestyle | 1.00 (ref.) | 1.00 (ref.) | 1.00 (ref.) | 1.00 (ref.) |
| Medium healthy lifestyle | 0.64 (0.51, 0.81) | 0.88 (0.58, 1.33) | 0.91 (0.33, 2.50) | 1.35 (0.43, 4.21) |
| Healthy lifestyle | 0.57 (0.41, 0.81) | 0.65 (0.37, 1.16) | 0.42 (0.08, 2.17) | 0.65 (0.08, 5.40) |
| $P_{trend}$ | <0.001 | 0.150 | 0.290 | 0.920 |
| $P_{interaction}$ | 0.231 | 0.270 | 0.930 | <0.001 |
| RERI | -0.89 (-1.59, -0.19) | -0.67 (-1.70, 0.36) | -0.07 (-1.49, 1.35) | 0.53 (-1.24, 2.30) |

-, Due to the small sample size, this value is not of reference significance. Model adjusted for age, sex, healthy lifestyle score, body mass index, the number of metabolic components traits, and prevalence of CVD, CBVD, CKD, diabetes, cancer and hypertension. CVD, cardiovascular disease; CBVD, cerebrovascular disease; CKD, chronic kidney disease; HR, hazard ratio; CI, confidence interval; MetS, metabolic syndrome; NHANES, National Health and Nutrition Examination Survey; RERI, relative excess risk due to interaction.

**Supplementary Table 21. Weighted and unweighted PAF for each lifestyle factors across social environments**

| Domains | UK Biobank | | | US NHANES | | |
|---|---|---|---|---|---|---|
| | Unweighted PAF | Communality | Weighted PAF | Unweighted PAF | Communality | Weighted PAF |
| Favorable social environments | | | | | | |
| Cigarette smoking | -0.348 | 0.498 | -0.174 | -0.163 | 0.225 | -0.137 |
| Alcohol consumption | -0.021 | 0.490 | -0.011 | -0.063 | 0.030 | -0.053 |
| Physical activity | -0.024 | 0.006 | -0.012 | -0.038 | 0.397 | -0.032 |
| Diet | 0.028 | 0.006 | 0.014 | -0.027 | 0.348 | -0.023 |
| Overall weighted PAF | | | -0.183 | | | -0.245 |
| Medium social environments | | | | | | |
| Cigarette smoking | -0.359 | 0.474 | -0.225 | -0.189 | 0.372 | -0.138 |
| Alcohol consumption | -0.024 | 0.476 | -0.015 | -0.040 | 0.174 | -0.029 |
| Physical activity | -0.039 | 0.000 | -0.024 | -0.013 | 0.165 | -0.010 |
| Diet | -0.023 | 0.051 | -0.015 | -0.081 | 0.289 | -0.059 |
| Overall weighted PAF | | | -0.278 | | | -0.236 |
| Unfavorable social environments | | | | | | |
| Cigarette smoking | -0.338 | 0.435 | -0.206 | -0.252 | 0.350 | -0.182 |
| Alcohol consumption | -0.017 | 0.431 | -0.010 | -0.026 | 0.319 | -0.019 |
| Physical activity | -0.014 | 0.005 | -0.009 | -0.013 | 0.073 | -0.009 |
| Diet | -0.013 | 0.129 | -0.008 | -0.075 | 0.257 | -0.055 |
| Overall weighted PAF | | | -0.234 | | | -0.265 |

Model adjusted for age, sex, body mass index, the number of metabolic components traits, and prevalence of CVD, CBVD, CKD, diabetes, cancer and hypertension. Weighted PAF was calculated after considering the overlap between risk factors. PAF, population attributable fraction; NHANES, National Health and Nutrition Examination Survey.

**Supplementary Table 22. The joint effect of social environments and lifestyle on all-cause and cause-specific mortality among adults with MetS in UK Biobank cohort**

| | All-cause mortality | CVD mortality | CBVD mortality | CKD mortality |
|---|---|---|---|---|
| | HR (95%CI) | HR (95%CI) | HR (95%CI) | HR (95%CI) |
| Favorable social environments and healthy lifestyle | 1.00 (ref.) | 1.00 (ref.) | 1.00 (ref.) | 1.00 (ref.) |
| Favorable social environments and medium lifestyle | 1.18 (1.05, 1.34) | 1.37 (1.09, 1.73) | 1.01 (0.65, 1.57) | 1.51 (0.75, 3.03) |
| Favorable social environments and unhealthy lifestyle | 1.38 (1.22, 1.56) | 1.52 (1.20, 1.92) | 0.85 (0.52, 1.38) | 2.26 (1.15, 4.42) |
| Medium social environments and healthy lifestyle | 1.03 (0.90, 1.19) | 1.19 (0.91, 1.56) | 0.82 (0.48, 1.39) | 1.54 (0.71, 3.34) |
| Medium social environments and medium lifestyle | 1.24 (1.10, 1.40) | 1.51 (1.20, 1.89) | 1.22 (0.80, 1.86) | 2.12 (1.09, 4.12) |
| Medium social environments and unhealthy lifestyle | 1.70 (1.52, 1.91) | 1.95 (1.57, 2.43) | 1.34 (0.88, 2.04) | 2.65 (1.38, 5.06) |
| Unfavorable social environments and healthy lifestyle | 1.54 (1.34, 1.77) | 1.88 (1.45, 2.43) | 0.95 (0.54, 1.64) | 2.70 (1.31, 5.56) |
| Unfavorable social environments and medium lifestyle | 1.80 (1.60, 2.02) | 2.17 (1.75, 2.70) | 1.65 (1.10, 2.49) | 2.97 (1.56, 5.65) |
| Unfavorable social environments and unhealthy lifestyle | 2.50 (2.24, 2.79) | 3.06 (2.49, 3.77) | 1.83 (1.23, 2.71) | 3.94 (2.10, 7.40) |
| $P_{trend}$ | <0.001 | <0.001 | <0.001 | <0.001 |

Model adjusted for age, sex, healthy lifestyle score, body mass index, the number of metabolic components traits, and prevalence of CVD, CBVD, CKD, diabetes, cancer and hypertension. Participants were grouped into nine groups according to social environments (unfavorable, medium and favorable) and healthy lifestyle score (unhealthy; medium; and healthy) with those with favorable social environments and healthy lifestyle as the reference group. CVD, cardiovascular disease; CBVD, cerebrovascular disease; CKD, chronic kidney disease; HR, hazard ratio; CI, confidence interval; MetS, metabolic syndrome.

**Supplementary Table 23. The joint effect of social environments and lifestyle on disease incidence among adults with MetS in UK Biobank cohort**

| | CVD incidence | CBVD incidence | CKD incidence |
|---|---|---|---|
| | HR (95%CI) | HR (95%CI) | HR (95%CI) |
| Favorable social environments and healthy lifestyle | 1.00 (ref.) | 1.00 (ref.) | 1.00 (ref.) |
| Favorable social environments and medium lifestyle | 0.99 (0.92, 1.07) | 1.12 (0.96, 1.30) | 1.17 (1.02, 1.34) |
| Favorable social environments and unhealthy lifestyle | 1.13 (1.04, 1.23) | 1.05 (0.89, 1.24) | 1.28 (1.12, 1.47) |
| Medium social environments and healthy lifestyle | 0.99 (0.91, 1.09) | 1.20 (1.01, 1.43) | 1.11 (0.95, 1.29) |
| Medium social environments and medium lifestyle | 1.12 (1.04, 1.21) | 1.18 (1.01, 1.38) | 1.23 (1.08, 1.40) |
| Medium social environments and unhealthy lifestyle | 1.21 (1.12, 1.31) | 1.30 (1.11, 1.52) | 1.39 (1.22, 1.58) |
| Unfavorable social environments and healthy lifestyle | 1.15 (1.05, 1.26) | 1.25 (1.04, 1.50) | 1.36 (1.17, 1.59) |
| Unfavorable social environments and medium lifestyle | 1.28 (1.19, 1.38) | 1.47 (1.26, 1.71) | 1.44 (1.26, 1.64) |
| Unfavorable social environments and unhealthy lifestyle | 1.46 (1.35, 1.57) | 1.80 (1.56, 2.08) | 1.67 (1.47, 1.89) |
| $P_{trend}$ | <0.001 | <0.001 | <0.001 |

Model adjusted for age, sex, healthy lifestyle score, body mass index, the number of metabolic components traits, and prevalence of CVD, CBVD, CKD, diabetes, cancer and hypertension. Prevalence of corresponding disease was not included for the analysis of diseases incidence. Participants were grouped into nine groups according to social environments (unfavorable, medium and favorable) and healthy lifestyle score (unhealthy; medium; and healthy) with those with favorable social environments and healthy lifestyle as the reference group. CVD, cardiovascular disease; CBVD, cerebrovascular disease; CKD, chronic kidney disease; HR, hazard ratio; CI, confidence interval; MetS, metabolic syndrome.

**Supplementary Table 24. The joint effect of social environments and lifestyle on all-cause and cause-specific mortality among adults with MetS in US NHANES cohort**

| | All-cause mortality | CVD mortality | CBVD mortality | CKD mortality |
|---|---|---|---|---|
| | HR (95%CI) | HR (95%CI) | HR (95%CI) | HR (95%CI) |
| Favorable social environments and healthy lifestyle | 1.00 (ref.) | 1.00 (ref.) | 1.00 (ref.) | - |
| Favorable social environments and medium lifestyle | 1.06 (0.83 ,1.36) | 1.22 (0.81 ,1.83) | 1.15 (0.42 ,3.12) | - |
| Favorable social environments and unhealthy lifestyle | 1.40 (1.07 ,1.83) | 1.05 (0.69 ,1.60) | 1.49 (0.55 ,3.99) | - |
| Medium social environments and healthy lifestyle | 1.27 (0.93 ,1.75) | 0.84 (0.47 ,1.48) | 1.72 (0.57 ,5.15) | - |
| Medium social environments and medium lifestyle | 1.79 (1.40 ,2.27) | 1.99 (1.33 ,2.98) | 2.04 (0.78 ,5.29) | - |
| Medium social environments and unhealthy lifestyle | 2.20 (1.70 ,2.84) | 1.56 (1.03 ,2.34) | 1.52 (0.56 ,4.11) | - |
| Unfavorable social environments and healthy lifestyle | 1.90 (1.29 ,2.79) | 1.33 (0.71 ,2.48) | 1.42 (0.34 ,5.93) | - |
| Unfavorable social environments and medium lifestyle | 2.28 (1.71 ,3.05) | 1.80 (1.14 ,2.82) | 1.59 (0.51 ,4.94) | - |
| Unfavorable social environments and unhealthy lifestyle | 3.54 (2.79 ,4.49) | 2.12 (1.40 ,3.22) | 1.48 (0.51 ,4.33) | - |
| $P_{trend}$ | 0.631 | <0.001 | 0.350 | - |

-, Due to the small sample size, this value is not of reference significance. Model adjusted for age, sex, healthy lifestyle score, body mass index, the number of metabolic components traits, and prevalence of CVD, CBVD, CKD, diabetes, cancer and hypertension. Participants were grouped into nine groups according to social environments (unfavorable, medium and favorable) and healthy lifestyle score (unhealthy; medium; and healthy) with those with favorable social environments and healthy lifestyle as the reference group. CVD, cardiovascular disease; CBVD, cerebrovascular disease; CKD, chronic kidney disease; HR, hazard ratio; CI, confidence interval; MetS, metabolic syndrome; NHANES, National Health and Nutrition Examination Survey.

**Supplementary Table 25. The joint effect of social environments and lifestyle on all-cause and cause-specific mortality among adults with MetS in UK Biobank cohort: using unweighted healthy lifestyle score**

| | All-cause mortality | CVD mortality | CBVD mortality | CKD mortality |
|---|---|---|---|---|
| | HR (95%CI) | HR (95%CI) | HR (95%CI) | HR (95%CI) |
| Favorable social environments and 3 or 4 healthy behaviors | 1.00 (ref.) | 1.00 (ref.) | 1.00 (ref.) | 1.00 (ref.) |
| Favorable social environments and 2 healthy behaviors | 1.06 (0.93, 1.20) | 1.20 (0.94, 1.54) | 1.21 (0.75, 1.96) | 1.21 (0.65, 2.26) |
| Favorable social environments and 0 or 1 healthy behaviors | 1.14 (1.01, 1.29) | 1.30 (1.03, 1.65) | 0.92 (0.56, 1.50) | 1.10 (0.60, 2.03) |
| Medium social environments and 3 or 4 healthy behaviors | 1.02 (0.89, 1.18) | 1.25 (0.96, 1.62) | 1.10 (0.66, 1.83) | 1.43 (0.76, 2.69) |
| Medium social environments and 2 healthy behaviors | 1.20 (1.06, 1.36) | 1.48 (1.17, 1.88) | 1.26 (0.79, 2.01) | 1.14 (0.62, 2.11) |
| Medium social environments and 0 or 1 healthy behaviors | 1.38 (1.22, 1.55) | 1.57 (1.25, 1.98) | 1.41 (0.89, 2.22) | 1.84 (1.04, 3.23) |
| Unfavorable social environments and 3 or 4 healthy behaviors | 1.58 (1.39, 1.80) | 1.92 (1.51, 2.45) | 1.66 (1.04, 2.67) | 1.91 (1.05, 3.47) |
| Unfavorable social environments and 2 healthy behaviors | 1.76 (1.57, 1.98) | 2.17 (1.74, 2.72) | 1.86 (1.20, 2.88) | 2.61 (1.50, 4.54) |
| Unfavorable social environments and 0 or 1 healthy behaviors | 2.11 (1.88, 2.36) | 2.61 (2.10, 3.25) | 1.72 (1.11, 2.68) | 2.19 (1.26, 3.82) |
| $P_{trend}$ | <0.001 | <0.001 | <0.001 | <0.001 |

Model adjusted for age, sex, healthy lifestyle score, body mass index, the number of metabolic components traits, and prevalence of CVD, CBVD, CKD, diabetes, cancer and hypertension. Participants were grouped into nine groups according to social environments (unfavorable, medium and favorable) and healthy lifestyle score (0 or 1; 2; and 3 or 4 points) with those with favorable social environments and three or four healthy lifestyle factors as the reference group. CVD, cardiovascular disease; CBVD, cerebrovascular disease; CKD, chronic kidney disease; HR, hazard ratio; CI, confidence interval; MetS, metabolic syndrome.

**Supplementary Table 26. The joint effect of social environments and lifestyle on disease incidence among adults with MetS in UK Biobank cohort: using unweighted healthy lifestyle score**

| | CVD incidence | CBVD incidence | CKD incidence |
|---|---|---|---|
| | HR (95%CI) | HR (95%CI) | HR (95%CI) |
| Favorable social environments and 3 or 4 healthy behaviors | 1.00 (ref.) | 1.00 (ref.) | 1.00 (ref.) |
| Favorable social environments and 2 healthy behaviors | 0.99 (0.91, 1.07) | 1.08 (0.91, 1.28) | 1.11 (0.97, 1.28) |
| Favorable social environments and 0 or 1 healthy behaviors | 1.06 (0.97, 1.14) | 1.13 (0.96, 1.34) | 1.02 (0.89, 1.16) |
| Medium social environments and 3 or 4 healthy behaviors | 1.03 (0.94, 1.12) | 1.21 (1.01, 1.45) | 1.02 (0.88, 1.18) |
| Medium social environments and 2 healthy behaviors | 1.10 (1.02, 1.20) | 1.29 (1.10, 1.53) | 1.23 (1.08, 1.40) |
| Medium social environments and 0 or 1 healthy behaviors | 1.14 (1.05, 1.23) | 1.23 (1.04, 1.45) | 1.13 (0.99, 1.29) |
| Unfavorable social environments and 3 or 4 healthy behaviors | 1.25 (1.14, 1.36) | 1.36 (1.14, 1.62) | 1.30 (1.13, 1.49) |
| Unfavorable social environments and 2 healthy behaviors | 1.25 (1.15, 1.36) | 1.69 (1.44, 1.98) | 1.42 (1.25, 1.62) |
| Unfavorable social environments and 0 or 1 healthy behaviors | 1.39 (1.28, 1.50) | 1.67 (1.43, 1.96) | 1.39 (1.22, 1.58) |
| $P_{trend}$ | <0.001 | <0.001 | <0.001 |

Model adjusted for age, sex, healthy lifestyle score, body mass index, the number of metabolic components traits, and prevalence of CVD, CBVD, CKD, diabetes, cancer and hypertension. Prevalence of corresponding disease was not included for the analysis of diseases incidence. Participants were grouped into nine groups according to social environments (unfavorable, medium and favorable) and healthy lifestyle score (0 or 1; 2; and 3 or 4 points) with those with favorable social environments and three or four healthy lifestyle factors as the reference group. CVD, cardiovascular disease; CBVD, cerebrovascular disease; CKD, chronic kidney disease; HR, hazard ratio; CI, confidence interval; MetS, metabolic syndrome.

**Supplementary Table 27. The joint effect of social environments and lifestyle on all-cause and cause-specific mortality among adults with MetS in US NHANES cohort: using unweighted healthy lifestyle score**

| | All-cause mortality | CVD mortality | CBVD mortality | CKD mortality |
|---|---|---|---|---|
| | HR (95%CI) | HR (95%CI) | HR (95%CI) | HR (95%CI) |
| Favorable social environments and 3 or 4 healthy behaviors | 1.00 (ref.) | 1.00 (ref.) | 1.00 (ref.) | - |
| Favorable social environments and 2 healthy behaviors | 0.84(0.64,1.10) | 0.84 (0.54, 1.33) | 1.14 (0.35, 3.72) | - |
| Favorable social environments and 0 or 1 healthy behaviors | 1.10(0.84,1.45) | 0.98 (0.64, 1.50) | 1.60 (0.53, 4.79) | - |
| Medium social environments and 3 or 4 healthy behaviors | 1.24(0.87,1.78) | 0.81 (0.44, 1.49) | 2.34 (0.68, 8.01) | - |
| Medium social environments and 2 healthy behaviors | 1.40(1.08,1.81) | 1.55 (1.01, 2.39) | 1.54 (0.49, 4.86) | - |
| Medium social environments and 0 or 1 healthy behaviors | 1.82(1.38,2.39) | 1.32 (0.86, 2.03) | 2.02 (0.68, 6.02) | - |
| Unfavorable social environments and 3 or 4 healthy behaviors | 1.85(1.24,2.74) | 1.30 (0.69, 2.42) | 1.57 (0.34, 7.29) | - |
| Unfavorable social environments and 2 healthy behaviors | 1.75(1.33,2.30) | 1.32 (0.81, 2.16) | 1.80 (0.52, 6.24) | - |
| Unfavorable social environments and 0 or 1 healthy behaviors | 3.00(2.33,3.86) | 1.86 (1.21, 2.87) | 1.49 (0.45, 5.00) | - |
| $P_{trend}$ | 0.203 | <0.001 | 0.310 | - |

-, Due to the small sample size, this value is not of reference significance. Model adjusted for age, sex, healthy lifestyle score, body mass index, the number of metabolic components traits, and prevalence of CVD, CBVD, CKD, diabetes, cancer and hypertension. Participants were grouped into nine groups according to social environments (unfavorable, medium and favorable) and healthy lifestyle score (0 or 1; 2; and 3 or 4 points) with those with favorable social environments and three or four healthy lifestyle factors as the reference group. CVD, cardiovascular disease; CBVD, cerebrovascular disease; CKD, chronic kidney disease; HR, hazard ratio; CI, confidence interval; MetS, metabolic syndrome; NHANES, National Health and Nutrition Examination Survey.

**Supplementary Table 28. The joint effect of social environments and lifestyle on all-cause and cause-specific mortality among adults aged≥ 40 years with MetS in US NHANES cohort**

| | All-cause mortality | CVD mortality | CBVD mortality | CKD mortality |
|---|---|---|---|---|
| | HR (95%CI) | HR (95%CI) | HR (95%CI) | HR (95%CI) |
| Favorable social environments and healthy lifestyle | 1.00 (ref.) | 1.00 (ref.) | 1.00 (ref.) | - |
| Favorable social environments and medium lifestyle | 0.86(0.65,1.13) | 0.84 (0.53, 1.32) | 1.15 (0.35, 3.73) | - |
| Favorable social environments and unhealthy lifestyle | 1.09(0.83,1.43) | 0.95 (0.62, 1.46) | 1.60 (0.53, 4.81) | - |
| Medium social environments and healthy lifestyle | 1.22(0.85,1.76) | 0.76 (0.41, 1.42) | 2.34 (0.68, 8.03) | - |
| Medium social environments and medium lifestyle | 1.41(1.08,1.84) | 1.55 (1.01, 2.39) | 1.53 (0.48, 4.83) | - |
| Medium social environments and unhealthy lifestyle | 1.78(1.34,2.35) | 1.29 (0.84, 1.99) | 1.90 (0.63, 5.73) | - |
| Unfavorable social environments and healthy lifestyle | 1.91(1.27,2.86) | 1.32 (0.71, 2.47) | 1.07 (0.19, 6.14) | - |
| Unfavorable social environments and medium lifestyle | 1.80(1.36,2.37) | 1.35 (0.82, 2.21) | 1.85 (0.53, 6.40) | - |
| Unfavorable social environments and unhealthy lifestyle | 2.99(2.30,3.89) | 1.77 (1.14, 2.74) | 1.58 (0.47, 5.27) | - |
| $P_{trend}$ | <0.001 | <0.001 | 0.330 | - |

-, Due to the small sample size, this value is not of reference significance. Model adjusted for age, sex, healthy lifestyle score, body mass index, the number of metabolic components traits, and prevalence of CVD, CBVD, CKD, diabetes, cancer and hypertension. Participants were grouped into nine groups according to social environments (unfavorable, medium and favorable) and healthy lifestyle score (healthy, medium and unhealthy) with those with favorable social environments and healthy lifestyle as the reference group. CVD, cardiovascular disease; CBVD, cerebrovascular disease; CKD, chronic kidney disease; HR, hazard ratio; CI, confidence interval; MetS, metabolic syndrome; NHANES, National Health and Nutrition Examination Survey.
