# Supplementary material for: Social environments and lifestyles linked to mortality and future health risks in metabolic syndrome: evidence from UK and US nationwide cohort studies
Source: BMJ Public Health. 2026 Aug 5;4(3):e004599. doi: 10.1136/bmjph-2025-004599 (PMC13475095; doi:10.1136/bmjph-2025-004599)
Supplement: Supplementary file 2 [file bmjph-4-3-s002.pdf]

Additional File 2

Study Cohorts

UK Biobank:

The UK Biobank (UKB) recruited over 500,000 volunteers aged 37-69 years between 2006 and 2010 (https://www.ukbiobank.ac.uk/). Individuals were invited to attend one of the 22 centers across England, Scotland, and Wales for baseline assessment. Written informed consent was obtained for the collection of questionnaire and biological data. UKB was conducted with ethical approval from the North West Multi-Center Research Ethics Committee of the UK (ref11/NW/0382). This research was conducted under UKB application number 107217.

US National Health and Nutrition Examination Survey:

The US National Health and Nutrition Examination Survey (NHANES) has been an ongoing annual survey since 1999, providing nationally representative samples of the non-institutionalized civilian resident population of the USA. Details have been described elsewhere[1]. The NHANES was approved by the NHANES institutional review board and National Center for Health Statistics Research ethics review board. In the NHANES study, individuals with MetS were defined using the same criteria as in the UKB study. Adults aged 20 years or older with MetS from the 1999 to 2018 cycles were included in our analysis.

Assessment of outcome variables

For all cause and cause-specific mortality, In the NHANES, deaths were ascertained through the National Death Index to Dec 31, 2019. In the UKB, deaths were ascertained through the death certificates held by the National Health Service Information Center (England and Wales) to November 30, 2022, and the National Health Service Central Register (Scotland) to November 30, 2021.

For incident non-fatal outcomes in UKB, CVD, CBVD and CKD cases were ascertained via electronic health records, including self-report, primary care, hospital

inpatient data, and death data, all mapped to ICD-10 codes, and the last linkage date was up to October 31, 2022 (England), August 31, 2022 (Scotland), and May 31, 2022 (Wales). At the time of analysis, hospital admission data were available until October 31, 2022 (England), August 31, 2022 (Scotland), and May 31, 2022 (Wales).

Assessment of social environments

For socioeconomic status, both cohorts assessed family income level, employment status, and education attainment; UKB additionally considered area-level income deprivation, while NHANES additionally considered food security[2]. For social capital, both cohorts considered race/ethnicity; UKB additionally considered living alone or with partners, social isolation, social support and social activity; NHANES additionally considered marital status[3]. For healthcare, both cohorts considered health care quality, while NHANES additionally considered health insurance coverage. For neighborhood and built environment, both cohorts considered accommodation stability; Additionally, UKB included factors such as natural environment, greenspace, and local crime rate[4]. Detailed cohort-specific definitions of social environment variables are provided below.

In NHANES

1) Employment: Participants were asked what type of work they had done in the last week and, if not working, the main reason why. Participants were classified as unemployed, except those who responded they were a student or retired were grouped with those reporting employment.
2) Poverty/income ratio: Participants were asked their family income and size, and the poverty/income ratio (PIR) is the ratio of family income to poverty. The Department of Health and Human Services poverty guidelines were used as the poverty measure to calculate this ratio. These guidelines are issued each year, in the Federal Register, for determining financial eligibility for certain federal programs. The guidelines vary by family size and geographic location.
3) Food security: Participants responded to the U.S. Food Security Survey Module questions (Bickel G, et al, 2000) whether: 1) they were worried if food would run out before there was money to buy more; 2) the food they bought didn't last and they didn't

have money to get food before there was money to buy more; 3) they couldn't afford to eat balanced meals; 4) they had cut the size of meals or skipped meals because there wasn't enough money for food; 5) if yes to #4, how often meals were cut or skipped; 6) they ate less than they felt they should because there was not enough money to buy food; 7) they were hungry but didn't eat because they couldn't afford food; 8) they lost weight because they didn't have enough money for food; 9) they did not eat for a whole day because there was not enough money for food; 10) if yes to #9, how often they did not eat for the whole day. Levels of food security were classified as follows: full food security, no affirmative responses; marginal food security, 1-2 affirmative responses; low food security, 3-5 affirmative responses; and very low food security, 6-10 affirmative responses[5]

4) Education level: Participants were asked for the highest grade or level of school they completed. The response categories are: less than 9th grade education, 9-11th grade education (includes 12th grade and no diploma), High school graduate/GED, some college or associates (AA) degree, and college graduate or higher.
5) Access to healthcare: Participants were asked if there is a place they usually go when sick or needing advice about health. If they answered "yes" or "there is more than one place" to this question, they were classified as having a routine place for healthcare. If yes, but the facility is a hospital emergency room, they were classified as not having a routine place for healthcare.
6) Health insurance: Participants were asked whether they are covered by health insurance or some other kind of health care plan. They are subsequently asked if covered by private insurance or several types of government insurance (Medicare, Medi-Gap, Medicaid, SCHIP, military health care, Indian Health Service, state-sponsored health plan, or other government insurance).
7) Marital status: Participants were asked whether they were married, widowed, divorced, separated, never married, or living with a partner. Those reporting marriage or living with a partner were grouped together.
8) Accommodation instability: Participants were asked if the home they are living in is owned, being bought, rented, or occupied by some other arrangement. A person was

considered to own the home even if they are still paying on a mortgage.

9) Race: Participants reported their race and ethnicity information. Mexican American, Other Hispanic, Non-Hispanic White, Non-Hispanic Black, Other Race - Including Multi-Racial.

In UK Biobank

1) Household income: Total household income before tax was obtained through questionnaires, and participants could choose an option from <£18 000 ($25 000; €21 000), £18 000-£30 999, £31 000-£51 999, £52 000-£100 000, >£100 000, do not know, or prefer not to answer.
2) Townsend deprivation index: Townsend deprivation index calculated immediately prior to participant joining UK Biobank. Based on the preceding national census output areas. Each participant was assigned a score corresponding to the output area in which their postcode is located.
3) Healthcare quality: Health score were included in Indices of Multiple Deprivation (IMD) scores, and it measures premature death and the impairment of quality of life by poor health. It considers both physical and mental health. The domain measures morbidity, disability and premature mortality but not aspects of behavior or environments that may be predictive of future health deprivation.
4) Education attainment: Participants reported their education qualifications as college or university degree; A levels, AS levels, or equivalent; O levels, GCSEs, or equivalent; CSEs or equivalent; NVQ, HND, HNC, or equivalent; other professional qualifications; none of the above (equivalent to less than high school diploma); or prefer not to answer (excluded as missing values).
5) Employment status: Participants were asked their employment status at baseline, we regrouped participants into two groups: employed (including those in paid employment or self-employed, retired, doing unpaid or voluntary work, or being full or part time students) and unemployed.
6) Living alone/ with partners: Participants were asked the following question: "Including yourself, how many people are living together in your household? Include those who usually live in the house such as students living away from home during term time,

partners in the armed forces or professions such as pilots, if you live alone, enter 1”.

7) Social support: Participants were asked the following question: "How often are you able to confide in someone close to you?" and participants could choose an option from almost daily, 2-4 times a week, about once a week, about once a month, once every few months, never or almost never, do not know, or prefer not to answer.
8) Social activity: Participants were asked the following question: "Which of the following do you attend once a week or more often? (You can select more than one)" and participants could choose from Sports club or gym, Pub or social club, Religious group, Adult education class, Other group activity, none of the above, or prefer not to answer.
9) Social isolation: Participants were asked the following question: "How often do you visit friends or family or have them visit you?" and participants could choose an option from almost daily, 2-4 times a week, about once a week, about once a month, once every few months, never or almost never, no friends/family outside household, do not know, or prefer not to answer.
10) Local crime rate: Crime score measures the rate of recorded crime in an area for four major crime types (violence, burglary, theft, and criminal damage) representing the risk of personal and material victimization at a small area level.
11) Accommodation instability: Participants were asked whether they own or rent the accommodation that they live in, and participants can choose an option from own outright (by you or someone in your household), Own with a mortgage, Rent - from local authority, local council, housing association, Rent - from private landlord or letting agency, pay part rent and part mortgage (shared ownership), Live in accommodation rent free, none of the above or prefer not to answer.
12) Natural environments remoteness: The percentage of the home location buffer classed as 'Natural Environment' in the Land Cover Map (LCM) 2007, and with home location data buffered at 1000m.
13) Greenspace: The percentage of the home location buffer classed as 'Greenspace', as a proportion of all land-use types, and with home location data buffered at 1000m.
14) Race: Participants reported their ethnic background in touchscreen questionnaires.

The procedures of weighting composite social environment score

First, we dichotomized the social environment variables into advantaged (0 point) and disadvantaged (1 point) categories[5, 6](**Supplementary Table S2**). Second, we obtained β coefficients from Cox regression models comparing all-cause mortality risk between disadvantaged and advantaged levels for each social environment variable separately. These models were adjusted for age, sex, lifestyle behaviors, body mass index (BMI), number of metabolic traits, and comorbidities (i.e., diabetes, hypertension, CVD, CBVD, CKD, and cancer). Finally, we calculated the weighted score by multiplying each binary social variable by its corresponding β coefficient, normalizing by the sum of coefficients, and scaling by the number of social variables.

Assessment of healthy lifestyle

1) Cigarette smoking (never, previous, current): Participants were asked about whether they have smoked over 100 cigarettes in life, never smoking was defined as smoking fewer than 100 cigarettes in life. Current smoking was defined as those who smoked >100 cigarettes in their lifetime and smoked cigarettes at the time of survey. Previous smoking was defined as having smoked >100 cigarettes during their lifetime and quit smoking before the time of the survey.
2) Alcohol drinking (<2 drinks/w, ≥2 drinks/w): Frequency and volume of current alcohol consumption were self-reported.
3) Physical activity (inactive, insufficiently active, active): Different assessment questions were used between the US and UK studies. In US NHANES, participants were categorized as inactive group (no leisure time physical activity), insufficiently active group (leisure-time moderate activity 1–5 times per week with metabolic equivalents ranging from 3–6 or leisure-time vigorous activity 1–3 times per week with metabolic equivalents >6), and active group (more leisure-time moderate or vigorous activity than above). In UK Biobank, participants were categorized as inactive group (no physical activity), insufficiently active group was defined as at least 150 min/week of moderate activity or 75 min/week of vigorous activity (or an equivalent combination), active physical activity group was defined as at least 300 min/week of moderate activity or 150 min/week of vigorous activity (or an equivalent combination).

4) Diet (healthy diet, unhealthy diet): In US NHANES, dietary quality was obtained from 24-hour dietary recalls and was assessed by healthy eating index (HEI) scores. The HEI-2010 was calculated for the 1999-2014 survey cycles, and HEI-2015 for the 2015-2018 survey cycles and the variable was directly provided by the original dataset. A healthy diet was defined as the health eating index in the top two fifths of distribution[7]. In UK Biobank, dietary information was obtained through questionnaires. According to a previous UK Biobank study, we evaluated dietary quality using a more recent dietary recommendation for cardiovascular health, which considered adequate consumption of fruit, vegetables, whole grains, fish, shellfish, dairy products, and vegetable oils and reduced consumption of refined grains, processed meats, unprocessed meats, and sugar sweetened beverages and a healthy diet was defined as meeting at least five items of the recommendations[8].

The procedures of calculating healthy lifestyle score

A healthy score included cigarette smoking (never, previous, and current), alcohol consumption (<2 drinks/week, ⩾ 2 drinks/week), physical activity (inactive, insufficiently active, and active), and diet (a healthy diet was defined as the Healthy Eating Index in the top two-fifths of distribution in NHANES and meeting at least five items of the recommendations in UKB, while an unhealthy diet did not meet the condition described for a healthy diet) according to a previous US NHANES study[9]. For each lifestyle factor, we assigned 1 point for a healthy level and 0 points for an unhealthy level. The definition of each lifestyle factor was based on the above description. Never smoking, <2 drinks/week, active physical activity and healthy diet were identified as healthy lifestyle. Although this simple additive method has been used widely[10-12], the underlying assumption is that the associations between different lifestyle factors and the outcome were identical, which might not be true. The healthy lifestyle scores ranged from 0 to 4, with higher scores indicating higher adherence to healthy lifestyle, and were subsequently categorized as favorable (3 or 4 healthy lifestyle factors), intermediate (2 healthy lifestyle factors) and unfavorable (0 or 1 healthy lifestyle factors) lifestyles.

Weighted Healthy Lifestyle Score

Since multiple lifestyle factors are interrelated and are associated with mortality and

morbidity, we constructed a weighted healthy lifestyle score including cigarette smoking, alcohol consumption, physical activity, and diet according to previous studies and that coincided with recommendations from the World Health Organization, accounting for varied magnitudes of the associations between different lifestyle factors and outcomes. For each lifestyle factor, we assigned 1 point for a healthy level and 0 points for an unhealthy level, and β coefficients for each lifestyle factor were estimated from the Cox regression model for all-cause mortality with adjustment of age, sex, body mass index (BMI), social environments, the number of MetS component traits and prevalence of comorbidities (diabetes, hypertension, cardiovascular disease, chronic kidney disease, CBVD and cancer). The weighted healthy lifestyle score was calculated as follows: the binary lifestyle factor was multiplied by the β coefficient, summed up, divided by the sum of β coefficients, and then multiplied by the count of lifestyle factors. The higher weighted scores indicating healthier lifestyles. Participants were grouped into tertiles: the bottom tertile represented the unhealthy lifestyle group, the middle tertile represented the medium lifestyle group, and the top tertile represented the healthy lifestyle group.

Reference

1. Statistics N.C.f.H. NHANES survey methods and analytic guidelines. 2023 [cited 2023 08.04]; Available from: https://wwwn.cdc.gov/Nchs/Nhanes/AnalyticGuidelines.aspx.
2. Zhong J., Zhang Y., Zhu K., et al. Associations of social determinants of health with life expectancy and future health risks among individuals with type 2 diabetes: two nationwide cohort studies in the UK and USA. Lancet Healthy Longev, 2024; 5(8): e542-e551. doi: 10.1016/s2666-7568(24)00116-8.
3. Muennig P., Cohen A.K., Palmer A., et al. The relationship between five different measures of structural social capital, medical examination outcomes, and mortality. Soc Sci Med, 2013; 85: 18-26. doi: 10.1016/j.socscimed.2013.02.007.
4. Xu J., Liu N., Polemiti E., et al. Effects of urban living environments on mental health in adults. Nat Med, 2023; 29(6): 1456-1467. doi: 10.1038/s41591-023-02365-w.
5. Bundy J.D., Mills K.T., He H., et al. Social determinants of health and premature death among adults in the USA from 1999 to 2018: a national cohort study. Lancet Public Health, 2023; 8(6): e422-e431. doi: 10.1016/s2468-2667(23)00081-6.
6. Zhao Y., Li Y., Zhuang Z., et al. Associations of polysocial risk score, lifestyle and genetic factors with incident type 2 diabetes: a prospective cohort study. Diabetologia, 2022; 65(12): 2056-2065. doi: 10.1007/s00125-022-05761-y.
7. Li Y., Schoufour J., Wang D.D., et al. Healthy lifestyle and life expectancy free of cancer, cardiovascular disease, and type 2 diabetes: prospective cohort study. Bmj, 2020; 368: l6669. doi: 10.1136/bmj.l6669.
8. Said M.A., Verweij N.,van der Harst P. Associations of Combined Genetic and Lifestyle Risks With Incident Cardiovascular Disease and Diabetes in the UK Biobank Study. JAMA Cardiol, 2018; 3(8): 693-702. doi: 10.1001/jamacardio.2018.1717.
9. Ford E.S., Zhao G., Tsai J., et al. Low-risk lifestyle behaviors and all-cause mortality: findings from the National Health and Nutrition Examination Survey III Mortality Study. Am J Public Health, 2011; 101(10): 1922-9. doi: 10.2105/ajph.2011.300167.
10. Zhang Y., Pan X.F., Chen J., et al. Combined lifestyle factors and risk of incident type 2 diabetes and prognosis among individuals with type 2 diabetes: a systematic review and meta-analysis of prospective cohort studies. Diabetologia, 2020; 63(1): 21-33. doi: 10.1007/s00125-019-04985-9.
11. Zhang Y.B., Pan X.F., Chen J., et al. Combined lifestyle factors, all-cause mortality and cardiovascular disease: a systematic review and meta-analysis of prospective cohort studies. J Epidemiol Community Health, 2021; 75(1): 92-99. doi: 10.1136/jech-2020-214050.
12. Zhang Y.B., Pan X.F., Chen J., et al. Combined lifestyle factors, incident cancer, and cancer mortality: a systematic review and meta-analysis of prospective cohort studies. Br J Cancer, 2020; 122(7): 1085-1093. doi: 10.1038/s41416-020-0741-x.

STROBE Checklist

	Item No	Recommendation	Page No
**Title and abstract**	1	(*a*) Indicate the study's design with a commonly used term in the title or the abstract	1
		(*b*) Provide in the abstract an informative and balanced summary of what was done and what was found	2
**Introduction**			
Background/rationale	2	Explain the scientific background and rationale for the investigation being reported	3,4
Objectives	3	State specific objectives, including any prespecified hypotheses	4
**Methods**			
Study design	4	Present key elements of study design early in the paper	5
Setting	5	Describe the setting, locations, and relevant dates, including periods of recruitment, exposure, follow-up, and data collection	5
Participants	6	(*a*) Give the eligibility criteria, and the sources and methods of selection of participants. Describe methods of follow-up	5
		(*b*) For matched studies, give matching criteria and number of exposed and unexposed	-
Variables	7	Clearly define all outcomes, exposures, predictors, potential confounders, and effect modifiers. Give diagnostic criteria, if applicable	5-8, Additional files 2
Data sources/ measurement	8*	For each variable of interest, give sources of data and details of methods of assessment (measurement). Describe comparability of assessment methods if there is more than one group	5-8, Additional files 2
Bias	9	Describe any efforts to address potential sources of bias	9-10
Study size	10	Explain how the study size was arrived at	11

STROBE Checklist

	Item No	Recommendation	Page No
Quantitative variables	11	Explain how quantitative variables were handled in the analyses. If applicable, describe which groupings were chosen and why	8
Statistical methods	12	(*a*) Describe all statistical methods, including those used to control for confounding	8-10
		(*b*) Describe any methods used to examine subgroups and interactions	9
		(*c*) Explain how missing data were addressed	Supplementary Figure S1
		(*d*) If applicable, explain how loss to follow-up was addressed	Supplementary Figure S1
		(*e*) Describe any sensitivity analyses	9-10
**Results**			
Participants	13*	(a) Report numbers of individuals at each stage of study—eg numbers potentially eligible, examined for eligibility, confirmed eligible, included in the study, completing follow-up, and analysed	11
		(b) Give reasons for non-participation at each stage	Supplementary Figure S1
		(c) Consider use of a flow diagram	Supplementary Figure S1
Descriptive data	14*	(a) Give characteristics of study participants (eg demographic, clinical, social) and information on exposures and potential confounders	11
		(b) Indicate number of participants with missing data for each variable of interest	Supplementary Table S5
		(c) Summarise follow-up time (eg, average and total amount)	11
Outcome data	15*	Report numbers of outcome events or summary measures over time	11
Main results	16	(*a*) Give unadjusted estimates and, if applicable, confounder-adjusted estimates and their precision (eg, 95% confidence interval). Make clear which confounders were adjusted for and why they were included	11-13

STROBE Checklist

	Item No	Recommendation	Page No
		(*b*) Report category boundaries when continuous variables were categorized	5
		(*c*) If relevant, consider translating estimates of relative risk into absolute risk for a meaningful time period	-
Other analyses	17	Report other analyses done—eg analyses of subgroups and interactions, and sensitivity analyses	12-13
**Discussion**			
Key results	18	Summarise key results with reference to study objectives	14
Limitations	19	Discuss limitations of the study, taking into account sources of potential bias or imprecision. Discuss both direction and magnitude of any potential bias	17
Interpretation	20	Give a cautious overall interpretation of results considering objectives, limitations, multiplicity of analyses, results from similar studies, and other relevant evidence	14-16
Generalisability	21	Discuss the generalisability (external validity) of the study results	14-16
**Other information**			
Funding	22	Give the source of funding and the role of the funders for the present study and, if applicable, for the original study on which the present article is based	19

*Give information separately for exposed and unexposed groups.

Note: An Explanation and Elaboration article discusses each checklist item and gives methodological background and published examples of transparent reporting. The STROBE checklist is best used in conjunction with this article (freely available on the Web sites of PLoS Medicine at http://www.plosmedicine.org/, Annals of Internal Medicine at http://www.annals.org/, and Epidemiology at http://www.epidem.com/). Information on the STROBE Initiative is available at http://www.strobe-statement.org
